# Bioinformatic Analysis of Complex In Vitro Fertilization Data and Predictive Model Design Based on Machine Learning: The Age Paradox in Reproductive Health

**DOI:** 10.3390/biology14050556

**Published:** 2025-05-16

**Authors:** Myrto A. Lantzi, Eleni Papakonstantinou, Dimitrios Vlachakis

**Affiliations:** 1 Laboratory of Genetics, Department of Biotechnology, School of Applied Biology and Biotechnology, Agricultural University of Athens, 11855 Athens, Greece; mylantzi@aua.gr (M.A.L.); eleni@aua.gr (E.P.); 2 University Research Institute of Maternal and Child Health and Precision Medicine, School of Medicine, National and Kapodistrian University of Athens, 11527 Athens, Greece; 3 Algorithms and Bioinformatics Group, Informatics Department, Faculty of Natural, Mathematical & Engineering Sciences, Strand Campus, King’s College, London WC2R 2LS, UK

**Keywords:** in vitro fertilization (IVF), live birth, cumulative live birth rate (CLBR), machine learning (ML), predictive model, perimenopause, late reproductive age

## Abstract

Since its inception in 1987, in vitro fertilization (IVF) has constituted a significant medical achievement in the field of fertility treatment, offering a viable solution to the challenge of infertility. The continuous evolution of assisted reproductive technology (ART) has brought its relationship with the rapidly developing field of artificial intelligence (AI), in particular with techniques such as machine learning (ML), a rapidly evolving area of specialization. In fact, it is responsible for significant developments in the field of precision medicine, as well as in preventive and predictive medicine. The analysis focuses on a large volume of clinical data and patient characteristics of those who underwent assisted reproduction treatments. Concurrently, the utilization of machine learning algorithms has facilitated the development and implementation of predictive models. The objective is to predict the success of treatments for external fertilization based on processed clinical data. This study encompasses statistical analysis techniques and artificial intelligence algorithms for the correlation of variables, such as patient characteristics, the history of pregnancies, and the clinical and embryological parameters. The analysis and creation of prognostic models were compared with the objective of identifying factors that influence the outcome of IVF treatments. The potential for implementing a predictive model in routine clinical practice was also investigated. The findings revealed trends and factors that warrant attention. Such findings may prompt questions regarding the impact of the patient’s age on treatment efficacy. Moreover, the value of developing a predictive model based entirely on patient data prior to the commencement of treatment was also investigated. This approach to the processing and utilization of clinical data demonstrates the potential for optimization and documentation of therapeutic procedures. The objective is to reduce costs and the emotional burden while increasing the success rate of IVF treatments.

## Simple Summary

In vitro fertilization (IVF) has been assisting millions of individuals and couples overcome infertility since its relatively recent introduction. The study analysed one of the largest real-world datasets in the field, containing clinical, embryological, and patient history data from over 1.5 million IVF attempts. Using machine learning methods, predictive models were developed to estimate the likelihood of a live birth based on the corresponding case’s characteristics. Statistical analyses and artificial intelligence algorithms were recruited to identify key factors that influence IVF outcomes. Our results confirmed the importance of age, among other variables, surprisingly noting the late reproductive age of a woman as a key target for further studies. Simultaneously, deep learning algorithms revealed that even models using only pre-treatment data can provide valuable predictions. These findings support the development of AI-powered tools that can help clinicians and patients make more informed, personalized decisions before starting IVF treatment. Ultimately, this approach aims to increase success rates, lower emotional and financial stress, and optimize fertility care.

## 1. Introduction

When considering innovative and out-of-this-world medical attainments, the conquest of infertility is one thing that undoubtedly comes to one’s mind, as many would have thought it could solely be an elusive dream. Fertility complications had been initially confronted by multiple natural fertilization attempts and human willpower. However, the concept of laboratory-based techniques being used for treating subfertility was roughly conceived in the 1930s and 1940s where experiments—as to in vitro oocyte maturation and fertilization outside of the human body—were performed, utilizing and testing either human, rabbit, or mouse gametes for fertilization under controlled conditions [1]. Further research led to in vitro fertilization (IVF) being discussed over its ethical clearance and indeed receiving the green light for clinical implementation. On 25 July 1978, the first IVF baby—Louise Brown—was born following a procedure performed by Robert Edwards, Patrick Steptoe, and Jean Purdy [2,3]. Since then, the term ‘assisted reproductive technology’ (ART) was at last established with IVF at the forefront.

The widespread adoption of IVF treatments and the establishment of ART clinical facilities and laboratories worldwide in the 1980s made IVF appear to be a medical procedure, owing to its proven positive impact on pregnancies and live deliveries. On that account, continuous advancements made in the ART field indicated a rise in the success rate of IVF, while they paved the way for the once experimental methods to be the conventional and the correlative techniques known today.

Specifically, IVF is the most accustomed reproductive technology where fertilization occurs by collecting and uniting sperm cells with oocytes on a petri dish, bypassing the natural process. It portrays the various stages of embryo development in vitro and then proceeds to the transfer of viable embryos into a potentially carrying uterus, with the primary goal of achieving a term live birth. The state-of-the-art approach, being implemented principally, proposes the induction of oocyte maturation by controlled ovarian stimulation (COS) and the then produced eggs’ retrieval. Simultaneously, sperm samples are collected and processed for in vitro fertilization to follow. Cultured blastocytes are assessed, transferred in the uterus, and supported for a chance at a fortunate pregnancy [4]. The consolidation of ART treatments for the past four decades has driven the development for sophisticated and intricate variations and techniques within the IVF domain, including intracytoplasmic sperm injection (ICSI), intrauterine insemination (IUI), gamete intrafallopian transfer (GIFT), zygote intrafallopian transfer (ZIFT), and more. These improvements emerge on the burgeoning need to further reshape the fertility treatment spectrum [5].

### 1.1. IVF Laboratory Procedure, Advancements, Techniques

Over the past four decades, the IVF laboratory procedure has evolved into an established lab protocol. While maintaining the base idea, a series of transformative advancements has shaped the modern IVF treatment globally practiced. Looking into detail, the journey began with retrieving mature oocytes from the mother and/or the individual providing the egg source. Initially, this step relied on tracking the natural menstrual cycle. The observation of the menstrual cycle aimed to track the luteal phase and determine the maturation state of oocytes based on hormonal level thresholds, like FSH and LH [6]. It was critical that actions were taken for inducing the maturation and the number of ovulatory follicles. Since then, the follicular, transvaginal, and endometrial monitoring has been accompanied by COS. This technique refers to injecting gonadotropins—once clomiphene (CC) and later human chorionic gonadotropin (hcG)—which, in conjunction with paracrine factors, such as GDF9, BMP-15, BMP-6, secreted by the oocytes themselves, was the first milestone in perfecting IVF methods. To further minimize premature ovulation, the use of gonadotropin release hormone agonist (GnRha), prior to ovarian stimulation by hcG, was established [7]. While the approach seemed optimal for patients, the fact that it led to an increased chance of subpar oocytes, along with high-quality ones and ovarian hyperstimulation syndrome (OHS) cases being reported, meant there was still room for improvement [6].

The amelioration of this step included the decrease in the number of injections required by introducing long-lasting gonadotropins, a huge field of interest resulting in purer, safer, and more efficacious hormone types. Stimulation is now administrated via pen injections, with gonadotropin schemes (rFSH, GnRH etc.) personalized for each patient. Optimization of ovarian response to the hormonal stimulation comes with individualized dosing based on the patients’ body weight and AMH serum levels. The anticipated upgrade of ovarian stimulation is the research and introduction of oral hormonal medication to streamline the process for both the patient and the embryologist–physician. Incorporating portable monitoring ultrasounds, ovarian reserve testing—static or dynamic—to identify the response to high doses of hcG and measuring the estradiol levels to minimize the need for constant blood draws has further enhanced the sufficiency of the procedure and has prevented complications like ovarian hyperstimulation syndrome (OHS) [6]. Refinements in stimulation protocols have involved the investigation of adjuncts in hormonal stimulation medication. Androgens, including DHEA and testosterone, have been studied for their potential to induce the number of follicles grown independently of gonadotropins. Additionally, growth factors have been introduced to further support the preantral follicle growth and maturation of oocytes. However, the benefits from such add-ons remain unsubstantiated. Similarly, heparin, compounds like aspirin and antioxidants such as vitamins, melatonin, zinc, and selenium or folic acid were tested but did not demonstrate efficiency in increasing live birth (LBR) or pregnancy (PR) rates. Apart from substance incorporation, obstetrics and gynecology procedures have been investigated for a key role in ameliorating IVF treatments. Endometrial scratching, originally employed as a treatment for endometriosis, has been promising in increasing implantation rates by promoting growth factor release. Accordingly, diagnostic tools like screening hysteroscopy have been proven to identify pathologies that may hinder IVF treatments and further enhance the outcome [8]. Patients’ hormone levels, like AMH, FSH, and AFC, serve as markers of the follicular growth level and ovulational state prior to the retrieval [6].

Proceeding to the actual retrieval, this sensitive microoperation needs quick handling, a stable pH level, a consistent temperature of 37 °C, minimal exposure to light, and heated needles [9]. The innovative introduction to microfluidics, the incorporation of ultrasonography, and a percutaneous needle alter the steps of the (once strictly) laparoscopic operation of follicle aspiration [4,7].

To improve the success of these steps, the development of techniques like in vitro maturation (IVM) was corollary. In IVM, it is possible to avoid exogenous gonadotropin administration and collect cumulus-enclosed immature oocytes from small antral follicles. This concept includes the reinforcement of the collected follicles by adding the paracrine factors GDF9, BMP, and CNP to delay cytoplasmic and nuclear maturation, respectively. Similarly, techniques like in vitro activation (IVA) and in vitro gametogenesis (IVG) aim to promote antral follicle growth or genesis in cases where there are little to no mature oocytes available [6].

Recent advancements involve enhancing mitochondrial function and understanding of the role of mtDNA in the quality of oocytes through supplements. Since mitochondria provide the cell with the energy required for multiple cell alternations, such as cytoplasmic and epigenome development, supplying the oocytes with coenzyme CoQ10 and performing an ooplasmic transfer has gained attention to whether it is the key for successful oocyte—and/or embryo—development [10].

Simultaneously, the sperm preparation takes place. The general protocol insists on a semen analysis, mandated by the World Health Organization (W.H.O.), as well as a test sperm preparation and a frozen sample back-up. The methods encountered for sperm manipulation are swim-up and microfluidics, which use a biocarbohydrate-buffered medium inside an incubator and discontinuous density gradient with a HEPES–MOPS-based medium buffer under air in centrifuge. The goal of semen processing is to eliminate seminal plasma, whose factors affect sperm function and fertility capacity when exposed over time [4]. It is now advised to run a serological status on frozen semen sample, before insemination, to determine the existence of viral load. Following those steps comes the assessment of both gametes available in controlled physicochemical conditions to avoid excess stress put to them [9].

During the insemination phase, sperm is introduced into a medium rich in glucose, compatible with the oocyte culture media for promoting fertilization while co-incubating. Regarding the start of embryo culture, the initial goal has always been to mimic the natural female reproductive environment by, initially, using tissue culture media—such as DMEM—or simple salt solutions, like Earle’s and T6. Both methods demand serum and supported solely cleavage-state embryos. To ensure the development of blastocyst in vitro, embryo-specific media were introduced, containing amino acids, nutrients, nucleotides, and another source of protein instead of serum—predominantly albumin. Serum was found to have adverse effects on the development, epigenetic state, and metabolic functioning of embryos. The breakthrough arose with the introduction of sequential media where the different formulations cover the diverse needs of the embryo as it goes through its developmental stages. This refinement resulted in G1 and G2 sequential mediums full of amino acids, vitamins, carbohydrates, antibiotics, phenol, and a changeable Ca:Mg ratio. Human serum is no longer used, while growth factors or cytokines are added. HSA solution, along with dextran, insulin, and transferrin, is also reported to be present in culture medias. As research progresses, the number of additional components incorporated in the culture media increases. For instance, hyaluronic acid—naturally found in the endometrium—or even glucose, growth factors, and AAs have been investigated for a potential beneficial contribution on pregnancy rates and embryo implantation. These incorporations are still awaiting adequate scientific validation [11]. However, one medium could never be suitable for all cultures; therefore, individual components are tailored to be optimized by patient, creating the perfect microenvironment for each case [4,6].

Another evolutionary establishment in the IVF method is the ability to monitor embryo development using time-lapse imaging. The assessment of cell division state and embryo morphology is possible without disturbing the incubation conditions or invading the embryo development [12]. Remarkably, when preimplantation genetic testing (PGT) was introduced, it pivoted the success rates of IVF, enabling the screening of genetic abnormalities and monogenic diseases. These advancements have only made the selection of higher-quality embryos successful and trustworthy [13].

After selection, embryos are either transferred or cryopreserved. Embryo transfer (ET) is a technique well established with little innovation since the first microoperation. It uses transvaginal and transabdominal ultrasounds, avoiding endometrial trauma or uterine contractions, along with specialized catheters, the fluidic volume and air bubble content of which plays a key role in prevention of ectopic pregnancy and possible embryo migration. Continuous testing has suggested that a soft catheter and a malleable stylet device with a transfer medium containing hyaluronan and hcG provides a higher likelihood of implantation occurrence. It is also advised to check for blood contamination of the catheter and to run a bioassay, prior to the transfer, to rule out possible embryo toxicity [4,6]. The actual biggest innovation in ET must be the single-embryo transfer, which has proved to minimize the multiple egg gestation and the pathologies that follow. It is noteworthy that running a frozen ET cycle seems to improve the pregnancy rates of the treatment, simply because the timing of the transfer can be aligned with the proper hormonal state of the carrier [6].

With that in mind, the cryopreservation of embryos and eggs has been a technique that boosts the success and popularity of IVF. Although cryopreservation and thawing initially posed risks to cells and embryos, subsequent improvement reintroduced freezing gametes, embryos, and ovarian tissues as a standard practice [7]. Cryopreservation is mainly performed in zwitterion-buffered and pH-stable cryoprotectant solutions. The advance came with the appliance of vitrification instead of slow freeze for blastocyst, eggs, and ovarian tissue where cells are rapidly cooled. This method secured a higher survival rate of embryos and outperformed slow freezing in conventional ART human embryo cryopreservation for both clinical pregnancies and live births [4,9].

The IVF laboratory procedure has continued to improve by presenting a variety of solutions for emerging obstacles. New technologies have even come to the forefront with the help of AI, where analyzing culture, gametes, and patients’ data, as well as mimicking natural environments, could provide accessible and precise embryo assessment–selection [14].

### 1.2. Merits and Demerits of ART Treatments

The forenamed practice has covered a groundbreaking number of arduous issues associated with fertility. While it is estimated that 10% of potential parents suffer from subfertility, IVF seems to offer multiple avenues for achieving conception [15]. Pregnancy is now possible, regardless of the obstacles in either men or women, related to sperm production, mobility and quality and female reproductive organ health and menstruation irregularity, respectively. Furthermore, possible peak fertility has been extended above 30 years old, elongating the age thresholds for parenthood. IVF seems revolutionary, allowing the creation of non-traditional families such as same-sex, non-binary, and single parents with or without carriers. On the contrary, conception and fertilization are separated. The time of conception can be decided based on the patients’ ease, provided by cryopreservation, while a multitude of pregnancies can occur from a single lab operation. IVF’s capacity to preserve fertility for those facing fertility-threatening medical conditions and treatment—including polycystic ovary syndrome (PCOS), primary ovarian insufficiency (POI), pelvic inflammatory disease (PID), endometriosis, and chemotherapy against cancer—represents a beacon of hope for inclusivity in parenthood [16]. Additionally, healthy offspring are provided through selection of embryos after PGT and screening for monogenic diseases [15].

However, it should not be forgotten that the development of the ART field is accompanied by considerations. Due to the financial cost of the process, even with partial insurance cover, the immediate success of the treatment is not ensured [17]. Equally important is the emotional impact of the treatment, hormonal medications, and the stress the treatment incites, all which can exert patients. Over and above that, several medical risks have emerged, including ovarian hyperstimulation syndrome (OHS), gestational diabetes, and pregnancy-induced hypertension [7]. Considering the propensity for multiple embryo transfers, pregnancy with twins and triplets is often inevitable, increasing the odds for preterm birth, low birth weight and small gestational size, health complications in infants, and higher chance of a cesarian section [18,19]. Looking into the demerits, protocols have yet to include regulated protocol add-ons, and in most cases, multiple cycles are advised. Patient inclusivity should be further assessed; many countries still serve heterosexual, married couples only, and some contraindications against circumvention of IVF treatments exist, preventing patients with rare medical conditions from entering the process [17].

### 1.3. Pathologies in IVF Babies

It is highly important that we acknowledge potential pathologies in IVF babies associated with the ART treatment procedure. The health risks of IVF have been a topic of discussion and research, although the majority of IVF offsprings are born with robust health and evade complications or morbidities. After the successful 1978 treatment, many IVF-conceived babies were born and have reached or surpassed the 40th year of their lives. Considering that the field of ART is relatively young, little research has been done to assess the health outcome of the offspring, but many suggest that there is mounting evidence of higher risk in some health states. The ART treatment is adept at ensuring an elevated embryo quality and reduced possibility of chromosomal–genetic abnormalities and monogenic diseases, in virtue of preimplantation genetic testing, such as PGT-A/M/S, creating an optimal genetic profile [15]. However, gestation does not conclude with embryo selection, and delving into the possible health risks requires elaborate research.

Several studies have underscored the possibility of multiple pregnancies and the health challenges that accompany them. A proclivity for preterm birth, prenatal mortality, and neonatal morbidity has been noted. Correspondingly, lower birth weight and a relatively small size for gestational age (SGA) has appeared at a heightened rate in IVF babies through multiple embryo transfer than single-ETs and at a much lower rate in naturally conceived infants [20]. Although a lower birth weight and a belated physical development along with SGA syndrome have been observed, most offspring tended to catch up in growth during their first years of life and have shown no major pathological features referring to physical development [21]. Apart from multiple gestation, such occurrence could be attributed to the choice of culture media [22], the actual reproductive morbidities of mothers consulting an ART treatment, or even the fact that ART indicated a rise in risk of obstetric complications, like preeclampsia and placenta previa [21,23]. It is worth noting that fresh cycle transfer may exhibit lower birth weight when compared to frozen-ET or natural gestation. It is significant to mention that an increased risk of inherent or de novo chromosomal abnormalities is present in ICSI treatments and can be ascribed to paternal infertility defects, particularly referring to the Y chromosome [21].

The epigenetic impact on infants has been a special topic of research. Since the environment plays a key role to the development, ART techniques like COS, the medication and drugs used, as well as the mothers’ psychological and hormonal state can affect the offspring epigenetically and cause imprinting and methylation defects. Relatively few investigations have shown evidence of potential subclinical hypothyroidism and euthyroid hyperthyrotropinemia in IVF babies when compared to control ones, with characteristic sustained and delayed TSH responses. Research has indicated that thyroid functions could be altered when exposed to the excessive hormonal medication of the mother, resulting in thyroid disorders and a potential epigenetic developmental morbidity. Such alternations could exert a latent influence on children’s gene expression and development [24].

On the contrary, meticulous examination has shed light in placental pathologies in IVF pregnancies. The increased thickness and weight of placentas and the presence of villitis of unknown etiology have been reported in IVF patients. These findings coincide with higher rates of maternal comorbidities, like fetal growth restriction (FGR) and preeclampsia, both of which are associated with adverse neonatal outcomes. Additionally, the elevated chance of a required cesarian section commands attention [25].

Initial research has focused on whether IVF methods increase the likelihood of congenital anomalies. While neonatal screening tests have yielded negative results and prove no concrete evidence of congenital defects, the issue remains unequivocal. To this effect, cardiovascular and neurodevelopmental abnormalities are also investigated but have yet to establish evidence for IVF conceived babies. Although research around birth malfunctions—such as imprinting disorders, syndromes like Angelman’s and Beckwith–Weidman, or even genitourinary defects—suggests a twofold higher prevalence among IVF–ICSI conceived children, when compared to naturally conceived ones, it has yet to overshadow the benefits of the treatment [7]. Notably, numerous studies have proven that no psychological–mental problems, long-term impact on their reproductive capability, nor a higher prevalence of medical and surgical illness are present, pointing to further pediatric research to reinforce the theories on pathology in IVF babies [23].

### 1.4. Assessment of ART Treatment Success

Overall, reproductive medicine has shown tremendous progress, enhancing pregnancy and childbearing. Indicators such as cumulative pregnancy rate (CPR) and cumulative live birth rate (CLBR) showed a profound rise [26]. For instance, CPR, which once was a single unit, surged to a 30% rate during the 1980s and now exceeds 50%, especially among women under 35 years old. Notably, multiple treatment cycles and the utilization of a donor seem to further increase the success rate [27]. Nonetheless, the indicators differ between clinics and countries and depend on patient-specific case factors. The type and cause of infertility noted, the age of the patient, the use of donor gametes, the treatment type used, and the number of previous treatment cycles are among the factors with a significant role in the treatment assessment. Markedly, we have amassed data spanning nearly three decades, referring to ART treatments at the clinic. The case entries exceed one million, each distinguished by various factors. These factors include the specific stimulation scheme used, the underlying reason for producing embryos or eggs, PGT occurrence, and detailed information regarding the number of eggs stored/fertilized/thawed and early outcomes. The richness of the entries made the dataset useful and complex, underscoring the relevance of precise record-keeping needed and the fluidity of ART treatment success.

Therefore, the ensemble of that many criteria could rarely be comprehensively controlled at once. This complexity is why ART is always in need of innovative alterations. The data we collected and processed indicate different success rates among different factors, the study of which can upgrade the main ideas of ART and hopefully result in evolutionary, nearly almighty achievements for overcoming subfertility. Taking it one step further, the concept of IVF is focused on creating the highest quality embryos for a guaranteed primary development. Contemplating improvement led us to the idea of shifting the focus from the embryo to the maternal and/or carrier, optimizing the environment for the fetus. The objective is a higher rate of fortunate further development and live births. This hypothesis seeks to further advance the possibilities in reproductive medicine.

### 1.5. Assisted Reproductive Technology: The Impact of Artificial Intelligence and Machine Learning Predictive Models

In the realm of analyzing data and generating new ideas, incorporating powerful tools like artificial intelligence (AI) seems promising. AI has integrated into the medical research and process optimization, and it has already offered progress in health care and diagnostics through wearables, speech recognition, and imaging algorithms [28]. Without question, it can be an effective appliance in precision medicine (PM). PM includes personalized treatment strategies based on an individual’s data, biomarkers, phenotype and genotype, lifestyle, and more [29]. Therefore, the concern rises to whether AI and ART methods can co-operate for treating conception incapability, case by case. To cite an instance of the invasion of AI in reproductive medicine, an ex vivo uterine environment (EVE) therapy was presented by Matt Kemp and Haruo Usuda referring to the creation of an artificial womb and placenta, currently using sheep as a model. This idea seems to initiate a step towards offering successful gestation. EVE is surely an innovative research pathway; however, it is accompanied by serious bioethical concerns [30].

Change has always been significantly accelerated by the state of technology. It has been revolutionary over the years and is currently evolving into what is known as web3.0, a more decentralized and semantic web thanks to artificial intelligence (AI), a more well-known innovation that is gradually permeating every aspect of daily life [28]. Machine learning (ML) and other AI tools appear to be the most similar to human intelligence in terms of how intelligence is not static but rather is improved through continuous training and knowledge consumption. Systems that use ML continuously retrain themselves using the provided data, increasing their performance and reliability over time [31]. Deep learning (DL), a subset of machine learning, was able to use artificial neural networks (ANNs), a massive network of data networks, for the only goal of analyzing patterns and producing predictions, with DL’s accuracy increasing with data import [32,33].

Healthcare management may now be handled differently as a result of the convergence of AI technologies with conventional medical practice and research. From keeping basic medical records to digitizing and increasing data complexity, which turns them into big metadata, medical care has changed significantly in even the most basic ways [34]. Currently, an individual case is distinguished by an abundance of characteristics that were previously potentially overlooked. The efficiency and consistency of medical care appear to be things that AI could handle, translate, and improve with ease [28]. As a result, single-person case evaluation and the use of tailored treatments appear to be feasible with the advent of personalized healthcare technology [29,35].

Herein lies the rise of predictive models. For big data handling and analysis, pattern recognition, statistical dependencies and relationships, and other purposes, a method utilizing DL and mathematical expressions has been developed [36]. By combining variables and indicators, these algorithms create a predictive model that can be used as a guide for ideal circumstances, well-informed decisions, and the development of exact treatment plans that are focused on the needs of the patient [37]. Even though data registration, analysis, algorithm development, modeling, and performance may seem like straightforward processes, a professional might find the big data meta-analysis itself to be excruciating [34]. In the field of clinical research, prediction models are being developed, particularly for data with less frequent target outcomes.

One area of medicine that has previously been studied for its advancements in AI technology is the reproductive health system. AI has developed its own area of analysis for reproductive health care, particularly in terms of reproductive technologies like IVF. Examples include oocyte and sperm quality, the assessment of embryo developmental potential, the classification of embryos, the monitoring of fertilization, and ploidy predictions. Statistical analysis, imaging, and assessment are just a few of the areas in which AI has made its mark in the field. Attempts to develop models that gauge the likelihood of a pregnancy are becoming more and more prevalent in terms of subfertility prognostic tools [31]. Single-case probability for successful IVF treatments can be calculated using patient, technical, and embryological data that can be easily included into the treatment plan for decision-making [38]. Predictive models, while sounding impressive, are still infrequently used in clinical practice because ongoing assessment and continuous data collection are still not common practices [36]. It presents a thorough, dependable, and useful algorithm when applied under the correct conditions.

With the potential improvements that predictive models could bring, the application of ART protocols could be made more efficient in terms of time, money, and waste by utilizing a variety of data sources, revealing patterns to forecast outcomes, complications, and risks, as well as diagnostic treatment patterns. It seeks to create a range of potential outcomes based only on each case and standardize, optimize, and digitalize the process that has improved in precision. Patients can be shielded from high-risk complications for both parents and children, as well as emotional strain and overmedication, using this additional tool for counseling. Recognizing the financial risk that one is willing to take is beneficial, especially in light of the expensive nature of ART procedures. This consultation provides a free-form, impartial, and objective probability based on different attributes. Consequently, the number of unsuccessful treatments decreases, and the success rates may match theoretical expectations.

Conversely, because human health is at stake, the strictures of medical procedures force the adoption of tried-and-true methods. It takes a thorough evaluation, retraining, and staff members who can register complex data to establish a predictive model [39]. The availability of data records is essential for the development of new models since it is imperative that the models should be trained using previous independent datasets. This is a concern because, up until now, medical histories have not been thorough or consistent. In addition, data registration has challenges and requires accurate labelling, fine tuning, quality control, and error rating. However, the risk of discrimination, invasion of privacy, or even diminished empathy increases because the data would be used in routine practice for these treatments.

Reproductive technology treatments have attempted to incorporate a number of prediction models; some have received FDA approval, but they remain unproven. The SART Patient Predictor and the Univfy prelIVF report are the most well-known and widely used predictive models created. They both use clinical case reports to identify characteristics that influence a positive IVF outcome [36]. Predictive models, like the one developed by Xu et al., are among the numerous active projects for estimating the likelihood of successful IVF treatments based on patient characteristics and variables encountered during treatment. The majority of existing models lack feature registrations, such as underlying conditions, hormonal states, etc., making them inconclusive [35,36,40].

Thus, following the trend, we made the decision to test and develop a prognostic model using the dataset we obtained, which included frequently reported characteristics, to provide a workable and relevant prediction system for popular ART treatments like IVF and DI. We decided to evaluate the effectiveness and significance of developing a prediction model for the successful outcome of IVF, which we defined as resulting in not only a pregnancy but also at least one live birth event. It was possible to predict the effectiveness of an IVF treatment for a specific patient without requiring them to have begun treatment by performing the modeling in two distinct landscapes: one aimed to make prognosis based on features referring to the whole process (in-cycle and pre-cycle characteristics), and the other based solely on predicting live birth by calculating pre-cycle, patient-focused features without evaluating the success based on embryological procedure variables.

In this paper, a plethora of datasets for IVF cases was meticulously analyzed, aiming to highlight the different variables of a successful childbearing. Studying the advances, data patterns, trends, and past results available, the aim was to uncover questions that arise. Since we are all still observing the late outcomes of IVF treatments in real life, we can only make assumptions for this—still ongoing—phase four of fertility treatment clinical trials. The goal is to surpass the obstacles and demerits of the existing fertility treatment methods applicable to each case by generating and proposing guidelines of the ideal conditions for gestation. While we decode how an individual hoping for parenthood could be ensured with a positive outcome, the bioethical challenges that arise should not be neglected.

## 2. Materials and Methods

### 2.1. Description of Data

The clinical datasets investigated encompass a substantial cohort of individuals opting for a fertility treatment at the clinic, throughout the past decades, in a timeframe of 27 years from 1991 to 2018, offering ample opportunities and options for multitude and comprehensive analyses. During those years, a sublime total of 1,546,070 fertilization attempts were conducted, with approximately 150,000–300,000 cases annually. In terms of demographic information, participants’ ages ranged from 18 to 50 years and consisted of various gender identities, since the subfertility factor and cause spanned from male sperm etiology to endometriosis, PCOS, tubal disease, and other female reproductive system disorders. Conspicuously, individuals participating had various assisted reproduction histories, having either reattempted an analogous treatment before, experienced previous gestations, or having commenced a treatment at the time. Indeed, the data present surfeit intricacy and inclusivity as each case is thoroughly characterized by an assembly of factors, including prior ART cycles; a provision of modalities, ART techniques, and process conditions; and a multitude of outcomes reported. This compilation of records, within the dataset, presents a wide array of perceptible conditions applicable to fertility treatment research.

### 2.2. Statistical Analysis

The acquired data were examined and stratified based on various clustering variables. Subsequently, case records were subjected to quantification and statistical analysis to reveal potential patterns. Initial analysis consisted of overall characteristic distribution analysis and tendencies among the individuals seeking treatment over more than 25 years of the clinic’s ART service. Furthermore, multivariate analysis, including Cox proportional hazard models and logistic regression, were employed to assess the impact of some variables on others. All statistical analysis demonstrated statistical dependence with a *p* value of <0.001, for the variables studied.

### 2.3. Predictive Model Design

#### 2.3.1. Data Processing–Wrangling

The first stage involved choosing wisely from all available data. We first chose to include datasets from later years to train models with cases that were far from the IVF establishment, with more modern, improved, and tested techniques, when IVF became more widely used and the procedures were optimized leading to fewer multiple gestations and complications. This decision was made from 27 years of recorded data. According to our preliminary bioinformatic analysis, the years 2010 and later showed the greatest improvement. As a result, datasets covering the years 2010–2018 were chosen for further processing.

From there, we ensured that the years we had chosen were complete and had a sufficient number of features and entries. Furthermore, coherence checks were performed on the datasets. After that, we processed the remaining 665,244 case reports. The merging of the various dataset files was the next step. This meant that to avoid misestimation and maintain coherence, features that were not present in every file were eliminated along with those that seemed to have more than 15% missing values. Additionally, all binary and numeric features were converted into categorical features. For example, features that used binary variables 1 and 0 were changed to “YES” and “NO”, and entries like 2 and 3 were changed to “2” and “3”. The processed merged file was split into two datasets, one with all selected features (30) and one with only pre-cycle features (18). This was the final step in the process.

#### 2.3.2. Data Registration

The two datasets were registered in the AI value-driven platform “DataRobot” to create two separate use cases in the platform workbench, one named “ar-2010-2018_complete features”, and the other “ar-2010-2018_pre-cycle features” (Table 1). DataRobot is a machine learning platform that deploys models derived from multiple algorithms and automates the procedure [41]. The complete feature list for each case is available in the Supplementary Material (Table S1).

#### 2.3.3. Modeling–Experiment Setting

Simultaneously, modeling was performed for both datasets by setting the prediction target as “Live birth occurrence”. The selection of the target was based on the features available and the biological significance of a live birth. The World Health Organization (WHO) defines a live birth as “the complete expulsion or extraction from its mother of a product of conception, irrespective of the duration of the pregnancy, which, after such separation, breathes or shows any other evidence of life (e.g., beating of the heart, pulsation of the umbilical cord or definite movement of voluntary muscles—whether or not the umbilical cord has been cut)” [42]. In the dataset the live birth occurrence variables, “YES” represented a successful live birth, and “NO” represented an adverse result; thus, the positive class was designated as “YES”.

The modeling mode for both use cases was set to comprehensive autopilot, meaning that utilizing the datasets, it could run as many repository model blueprints as possible, resulting in higher accuracy and an extended run time. The available algorithms could be run using different optimization metrics, and for our experiment, the area under the curve (AUC) seemed the most suitable for the structure of our data as a performance measure, as compared to bibliography. The goal was to develop models validated with an AUC score perceived as reliable, since an AUC score under 0.5 is considered to have poor predicting ability [43,44]. As for the training feature list, only informative features were included, meaning that only features passing a certain level of useful information would be added for training and measures for their impact. For both cases—complete feature and pre-cycle feature cases—all variables were calculated as impactful; thus, 30 and 18 features were used to train the model, respectively. The metric chosen was performed under stratified sampling, where the extremely large dataset was limited to 50,000 rows without lacking silhouette score estimation efficiency. Additionally, the technique for validation partition for model performance was based on cross validation, where the data were split into several subsets, defined as “folds”. These folds created a single model, while the remaining “unfolded” data were assigned for training. Concurrently, a fold called holdout that contained data not available for use by any model was chosen as the 20% of data and was used as final model performance estimation, only for the model selected to exclude selection bias.

The modeling workflow consisted of multiple different models based on different algorithm types. It works with either linear models, like L1, L2, and ElasticNet; or tree-based models, such as eXtreme Gradient Boosted Trees (XBoost), Random Forest, and GBM; and deep learning foundational models, like the Keras model. The comprehensive autopilot method manages to extract 59 models for each case, marking the most highly validated and biggest sample sized one as prepared and recommended for deployment. Each model is characterized by a blueprint, a machine learning pipeline showing all processing steps and algorithms recruited. Additionally, models calculate feature impacts, feature effects, and other meta-analysis variables, such as the ROC curve and lift chart.

The algorithms chosen for further studying and processing for both cases were:

eXtreme Gradient Boosted Trees Classifier with Early Stopping (learning rate = 0.02): An ensemble of machine learning decision tree models consequentially built where each precedent tree is used to improve and evaluate the next one until a final product is generated as a highly evaluated, powerful, and efficient prediction model. More hyperparameters are added in contrast with other conventional boosting tree algorithms and each newly generated tree is fitting the residual of prior trees and extending the prognostic ability. Adding the early stopping as a regularization setting overfitting is mitigated by establishing a stopping point in the tree training iterations [45,46,47].

Keras Deep Residual Neural Network Classifier using Training Schedule (3 Layers: 512, 64, 64 Units): The model runs with three-layer configurations while it vanishes gradient issues. Broad input data patterns are derived from the first layer (512 units), allowing learning refinement during the latter layers of 64 units. The model is based on dynamic training characterized by variable alterations and gradual increase of learning rate [33]. Concerning medical diagnostic technologies, neural network type algorithms are widely used in Datarobot-like platforms [48].

Notably, only informative features were included in the modeling process in both cases. However, in the complete feature case leakage was removed so that features which included values and information that were impossible to be known prior to the target outcome prediction would not affect feature impact calculations. From there, the chosen model was ready for deployment, and an application was generated. The application environment enabled the exploration of the parameters and analysis, as well as a questionnaire for a new individual patient’s case registration and immediate prediction results.

## 3. Results

### 3.1. Results from Statistical Analysis

**Figure 1:**
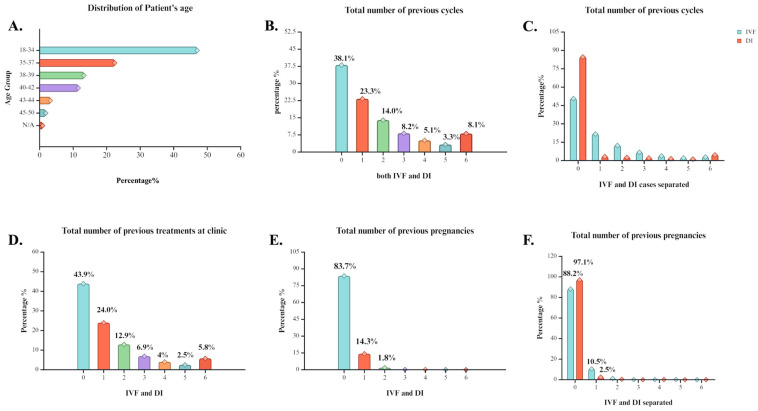
Statistical analysis of general history characteristics of cases that underwent ART treatment at the clinic between 1991 and 2018. (**A**) Percentage distribution of individuals across different age groups. Age clusters vary from 18–50 years old and N/A refers to individuals with their age not registered. (**B**) Percentage distribution of individuals based on the number of previous ART cycles reported. (**C**) Percentage distribution of individuals based on the number of previous IVF or DI cycles reported. (**D**) Percentage distribution of individuals based on the number of previous treatment procedures at the clinic. (**E**) Percentage distribution of individuals based on the number of previous pregnancy occurrence. (**F**) Percentage distribution of individuals based on the number of previous pregnancy occurrence separated by method induced. Created with BioRender.com.

The examination of demographics initially revealed the participant’s age distribution spanning from 18 to 25 years. Most cases appeared in the bracket of 18–34 years, followed by a gradual decline for the 35–50-year-old subgroups. The distribution suggests an apparent trend for pursuing childbearing during the human reproductive prime and a decreasing tendency in other age cohorts. Specifically, participants aged 18–34 years represented 47.0%, 35–37 years represented 22.4%, 38–39 years represented 13.2%, 40–42 years represented 11.5%, and 45–50 years represented 1.8%, respectively (Figure 1A). Regarding the frequency of ART treatment encounters, the individuals engaging in an analogous procedure seemed to gradually increase since 2010. In former years, treatment-seeking behavior remained stable.

Delving into the insights of the cases, the records of the ART history of each participant were obtained. Among the total of 1,546,070 treatment attempts, less than half had no prior experience with an ART procedure before, signifying that 38.1% of cases had zero previous IVF–DI cycles reported, while 23.3% of the cohort had undergone a treatment once. Noticeably, the percentage of individuals engaging in ongoing regiments exhibited a gradual decline, yet an evident group of cases (8.1%) exceeded six prior cycles (Figure 1B). Briefly focusing on the method used, IVF was more employed as the number of treatment cycles increased when compared to DI. Conversely, DI seemed to make a bolder appearance as the preferable technique for cases with more than five tries (Figure 1C). For patients with prior attempts at the clinic, the same pattern followed, albeit with higher prevalence of cases with no prior experience of a treatment from the clinic before (Figure 1D). Another useful report for investigating the outcomes was the presence of previous pregnancies. A crushing 83.7% of cases were attempting their first pregnancy (Figure 1E), but method-wise IVF was more frequent than DI among the few cases where previous pregnancies had been reported (Figure 1F).

**Figure 2:**
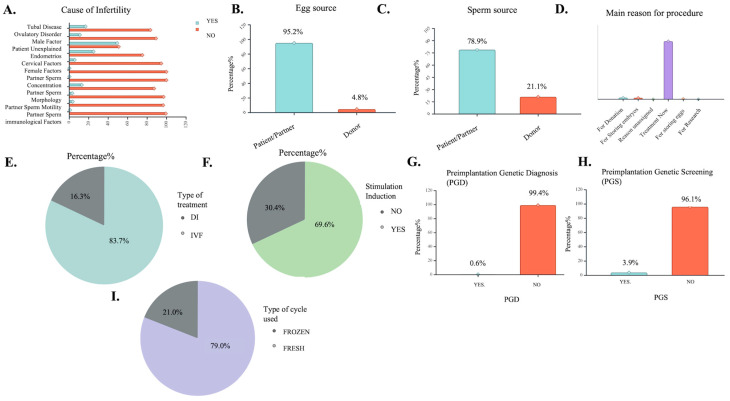
Statistical analysis of treatment insights at the clinic of cases that underwent ART treatment at the clinic between 1991 and 2018. (**A**) Distribution of presence of specific cause of infertility among the cases. (**B**) Percentage of cases’ option for egg source. (**C**) Percentage of cases’ option for sperm source. (**D**) Distribution of main reason for treatment assigned. (**E**) General treatment type and percentage of its execution among the cases. (**F**) Percentage of stimulation induction usage among the cases. (**G**) Occurrence of preimplantation genetic diagnosis. (**H**) Occurrence of preimplantation genetic screening. (**I**) Percentage of cases that used either a fresh or a frozen cycle during the treatment. Created with BioRender.com.

Upon scrutiny of the modalities employed in each case, IVF was favored for 83.7% of cases, while the remaining 16.3% was treated with a DI method (Figure 2E). Examining the impetus for treatment, a male subfertility factor was recorded as the main reason, followed by unexplained conditions, tubal disease, and ovulatory disorder of the treated patients (Figure 2A). It is noted that the primary objective of the procedure was immediate treatment—accounting for 94.4% of cases—while storing eggs and embryos were among the available treatment options (Figure 2D).

The striking subset of cases (95.2%) reported treatment involving the use of patient’s eggs (Figure 2B), while concurrently, 78.9% of total cases produced embryos through the insemination of eggs with patients’, sperm and 20.1% utilized sperm from a donor (Figure 2C). Significantly, a mere 0.2% of individuals acted as surrogates, and 0.7% assumed the role of embryo donors since the main reason for the treatment assigned was successful subfertility treatment. Moreover, adjuncts for stimulation were incorporated in the treatment protocol in over half the cases (69.6%) (Figure 2F). However, the specific type of ovulation induction was—in most instances—either not recorded (63.2%) or referred to gonadotrophins (29.2%). On the contrary, the cases where a preimplantation genetic diagnosis (PGD) was performed eased to 0.6% (Figure 2G) and, respectively, the preimplantation genetic screening (PGS) was conducted at a 3.9% rate (Figure 2H).

As far as the procedural insights are concerned, approximately 8 in 10 cases (79.9%) were treated immediately using a fresh cycle, with the remaining 21.1% opting for a frozen cycle ART protocol (Figure 2E). Embryological information included the number of oocytes and/or embryos retrieved, stored, thawed, mixed, microinjected, and transferred (Table 2).

**Figure 3:**
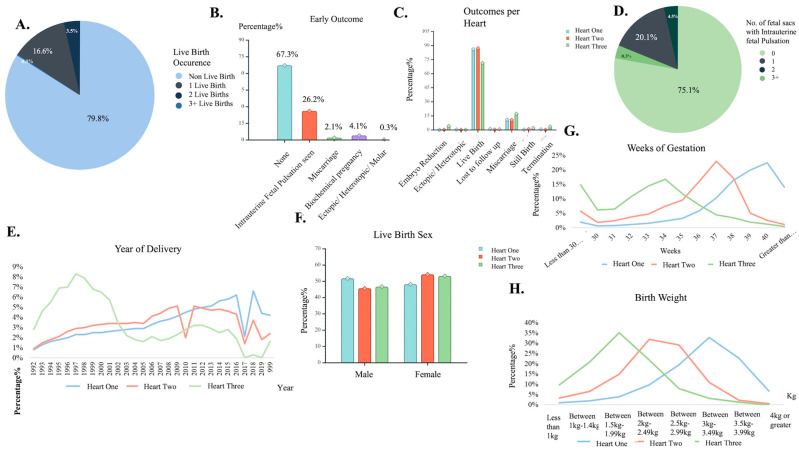
Deliveries: fetal sacs’ characteristic insights. (**A**) Number of live birth occurrence pie chart. (**B**) Early outcome distribution among cases. (**C**) Different outcome distribution based on hearts found. (**D**) Number of fetal sacs seen with a pulsation pie chart. (**E**) Live birth delivery occurrence of Heart found, by year. (**F**) Birth sex assignment distribution by hearts found. (**G**) Weeks of gestation occurrence distribution for the different number of hearts found. (**H**) Newborn weight measurement distribution for the different number of hearts found. Created with BioRender.com.

Out of the 1.5 million reported cases, more than 1.2 million (79.8%) resulted in non-live births, and successful outcomes were measured at a 20.2% success rate (Figure 3A). Multiple gestations occurred in 3.6% of treatments, with one successful live birth reported in 16.6% of cases, representing the most common outcome of successful treatments (Figure 3A). Furthermore, the early outcomes of treatments conducted at the clinic were meticulously documented. In more than two-thirds of cases (67.3%), no results were found during the examination, whereas 26.6% revealed intrauterine pulsations as the primary result. A lower rate of 2.1% of procedures led to miscarriages, and 4.1% were classified as biochemical pregnancies only. Only a few cases (0.3%) reported complications such as ectopic, heterotropic, or molar pregnancies (Figure 3B).

When examining the fetal sacs, 75.1% of total cases showed no observations of fetal sacs, regardless of the early outcome. Moreover, 20.1% of treatments reported only one fetal sac, representing the most common outcome of intrauterine pulsations observed. The remaining 4.5% and 0.3% reported two and more than three fetal sacs after treatment, respectively (Figure 3D). Overall, a successful outcome was more commonly characterized by one fetal sac and one live birth. However, a year-by-year analysis of cases revealed fluctuations. It was observed that single deliveries through IVF had a gradual increase, marking the departure from the once frequent occurrence of multiple gestations during fertility treatments. From 1991 to 2002, triplets were a common outcome, but advancements in ART treatments have steadily mitigated this risk. From 2003 to 2012, successful treatment mainly resulted in twin deliveries, and from 2012 and onward, a predominant trend of single live births emerged. Notably, quadruplet deliveries provided no adequate data for analysis (Figure 3E).

The data further verified that multiple gestations entailed complications (differentiations) such as shorter gestation times. Conversely, single deliveries exhibited no deviation from natural fertilization cases demonstrating a gestation timespan exceeding 38 weeks of gestation (Figure 3G).

Cases that advanced to the later stages of the procedure and reported fetal sacs were recorded for their birthing outcomes. Most of these cases culminated in live births, irrespective of birth order, with a slightly elevated occurrence of live births observed in second-born children than those being born first. Miscarriage was the most prevalent complication, while third-to-be-born children were more susceptible to experiencing other outcomes, including embryo reduction, termination, or stillbirth (Figure 3C). Most first-borns were designated male at birth, while more than half second- and third-born children were assigned as females at birth (Figure 3F). Accordingly, birth weight was documented for first-borns, typically weighing three or more kilograms, and second and third children exhibited a tendency for lower and riskier birth weights (Figure 3H).

All these data were easily interpreted and appeared helpful for identifying characteristics that contributed to a successful treatment, so it enabled the unveiling of questionable trends associated with varying outcomes, thus warranting further explanation and elucidation.

### 3.2. Interpretation of Results—Major Findings

Analysis revealed statistical dependence among variables, hinting that live birth occurrences may be influenced by the factors under consideration. As age appeared to be a significant factor, cases were stratified based on live births. As expected, the most successful group compromised patients aged 18–34, while those aged 35–37 years exhibited a comparable distribution between live and non-live births, albeit with an overall positive outcome. As the age increased, the proportion of failed attempts was nearly double the live births. Of note is that the 43–44 and 45–50 age groups demonstrated similar cumulative live birth rates (CLBR), but a higher unsuccessful live birth rate appeared for the 43–44-year-old group (Table 3).

Additionally, the presence of no or at least one previous cycle was associated with a favorable prognosis in live births. Conversely, a history of previous pregnancies emerged as a predictor of successful outcomes. Live births exhibited a higher percentage of elective single-embryo transfer interpreted in the protocol, and IVF was more prevalent in cases culminating in live births than in the non-live ones. The use of an egg donor and partner’s sperm were observed in higher frequencies in live birth cases (Table 4).

Similarly, clustering by occurrence, fresh cycles for performing ART demonstrated a higher percentage of CLBR compared with frozen cycles, noting that a greater number of fresh cycles were conducted overall. Conversely, frozen cycles presented a similar proportion of unsuccessful treatments (Table 5). Accordingly, most cases exhibiting favorable outcomes incorporated stimulation, thereby fueling further discourse on the efficacy of stimulants in IVF procedures (Table 5).

Overall, the incidence of live births reported increased when more oocytes were mixed, and more embryos were created and transferred (Table 6). As anticipated, live births exhibited an upward trend over time, presenting higher occurrences noted after 2005.

Regarding the etiology of subfertility, each identified reason, when present, seemed to impede and challenge a live birth occurrence. Nonetheless, the treatment appeared to overcome the subfertility cause, when associated with endometriosis, ovulatory disorder, and an unexplained reason (Table 7).

A preliminary guideline could be formulated based on every treatment characteristic associated with a positive prognosis. This framework would allow the overall procedure to be conducted with either IVF or DI depending on the subfertility case, as both methods demonstrate efficacy. Notably, conducting treatment on a fresh cycle during the patient’s initial or subsequent try, along with incorporation of stimulation induction, has emerged as a favorable prognostic protocol. Moreover, mixing more eggs and creating or transferring a larger number of embryos while adhering to the latter years’ protocols has yielded successful outcomes. The utilization of donor’s oocytes have provided a promising avenue for improving prognosis, although in cases of endometriosis, ovulatory disorder, or an undetectable infertility cause, the treatments with patients’ eggs appeared potent. It has been well established and confirmed that patients in the 18–34 age bracket could be confidently offered a potential successful treatment.

**Figure 4:**
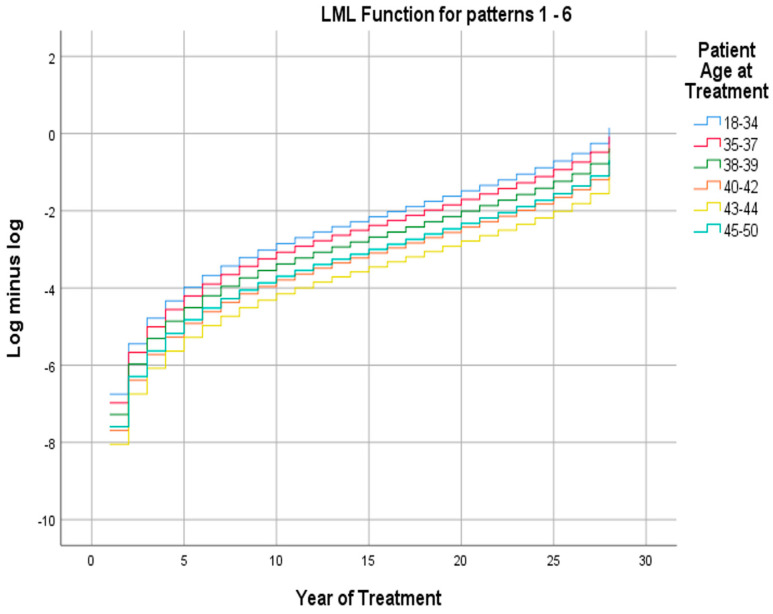
Statistical Analysis: a log-minus-log plot of the live birth occurrence for the year of treatment using as covariate the patient age at treatment, depicting CLBR in each age bracket throughout the years of IVF procedures at clinic.

Studying unpredictable outcomes, a log-minus-log plot of the live birth occurrence depicting the live birth occurrence over the year of treatments, with the patients’ age as covariate, was found to deviate from established literature. Conventionally, the cumulative live birth rate gradually diminishes with advancing age, particularly for women over 40 years old, where the decline is pronounced with each year of age increase, suggesting an age-dependent decline in IVF success. Employing a Cox proportional hazard model with the year of treatment as the time variable, live birth occurrence as the outcome, and the patient age at treatment as the covariate, a statistically significant model was demonstrated. The plotted data illustrate the possibility of a positive outcome in each age group. Following an expected trend, all age groups improved in CLBR over time with the 18–34 age bracket, exhibiting the best prognosis indicator. Ages 35–37 followed with a 20.1% less likelihood of a live birth, and ages 38–39 exhibited a corresponding decrease of 41.0%. The paradox arose in ages 45–50 displaying the fourth best probability with a 57.0% reduction in possibility of a positive outcome. Conversely, the 40–42 age group demonstrated a 61.0% probability of live births, and the least favorable outcomes were observed for the 43–44 age group, with a 72.8% reduction in probability (Figure 4).

### 3.3. Live Birth Predictive Models Based on Machine Learning

#### 3.3.1. Modeling for “ar-2010-2018_Complete Features”, Predictive Model for Live Birth Prognosis Based on All Features Available in the Dataset: Informative Features—Leakage Removed

Modeling: 59 possible models were generated for live birth occurrence target, with comprehensive autopilot and an AUC optimization metric, and based on informative features with stratified sampling and set for cross-validation and 20% holdout. The highly validated and 100% sample size algorithm was used for application development. The main model was compared to a sufficiently validated one but based on a completely different algorithm type and appeared with a 64% sample size (425,756 row usage) (Table 8).

**Figure 5:**
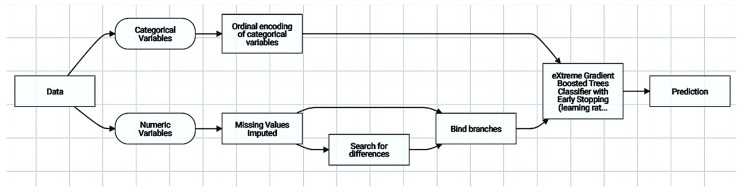
Blueprint extracted from the chosen algorithm of the “ar-2010-2018_complete features” dataset modeling. The blueprint explains the dataset’s pre-processing, post-processing, and algorithm deployment steps for model development.

The initial dataset’s pre-processing, post-processing, and algorithm deployment steps are all included in the blueprint, which explains the modeling process. Further data processing was therefore essential for the development of the model, and the procedures are digitalized below (Figure 5).

**Figure 6:**
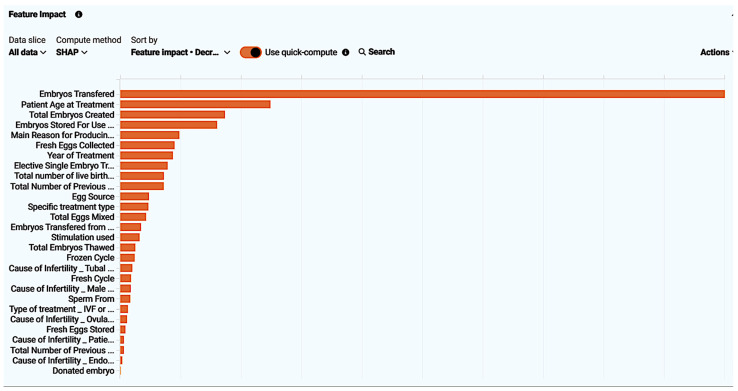
Feature impact of every feature present in the “ar-2010-2018_compete features” dataset visualized. Feature impact ranks all features based on their influence in the live birth occurrence.

The model explains which of the impactful features had a more key role in decision-making. These impacts were measured using training data. The feature impact was calculated and normalized to a 0–100 scale by the SHAP method. The findings suggest that features with a high impact in determining the live birth occurrence were 1. embryos transferred (raw score = 1.033, normalized score = 100%); 2. patient age at treatment (raw score = 0.257, normalized score = 24.84%); 3. total embryos created (raw score = 0.179, normalized score = 17.31%); 4. embryos stored for use by patients (raw score = 0.165, normalized score = 16.01%); 5. The main reason for producing embryos, storing eggs (raw score = 0.101, normalized score = 9.76%); 6. fresh eggs collected (raw score = 0.093, normalized score = 8.96%). More specifically, relative feature impact is demonstrated both as a chart (Figure 6) and table (Table 9) for easier analysis.

**Figure 7:**
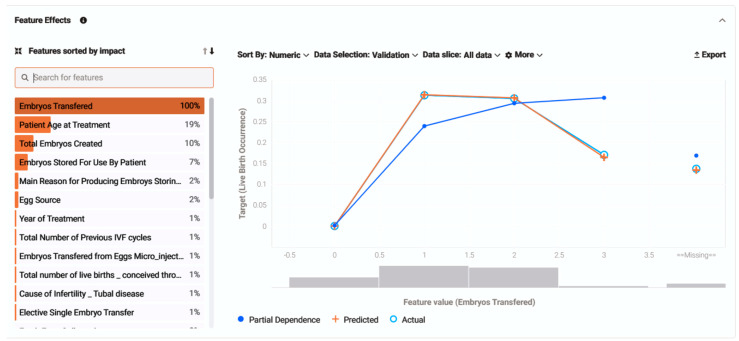
Feature effect visualized by displaying the most common values with the target, resulting in a partial dependence graph line (blue dot line), the average prediction for an individual feature value (orange crosses), and the actual target outcome for the individual feature values (light blue circles). In this figure “Embryos Transferred” feature effect is displayed. All feature effects refer to “ar-2010-2018_complete features” dataset modeling.

Addressing feature effect, the model managed to perform a calculation of how the prediction outcome can be affected by changes in each feature based on their impact. It determined the relationship between each feature individually and the target, without altering other feature values. The five features with the most significant impact, with a decreasing effect percentage, were: 1. embryos transferred; 2. patient age at treatment; 3. total embryos created; 4. embryos stored for use by patient; 5. the main reason for storing embryos, producing eggs. For reference, the chart below interrelates and displays one of the most effective values (embryos transferred) in correlation with the target, resulting in a partial dependence graph line (blue dots) explaining how changes in each unique feature regulate the prediction, unveiling their relationship. Simultaneously, orange crosses calculate an average prediction for an individual feature value while still focusing on the feature selected while leaving all other features stable. The light blue circles calculate the actual target outcome for the individual feature values and compare actual and predicted target values, helping evaluate model accuracy (Figure 7).

**Figure 8:**
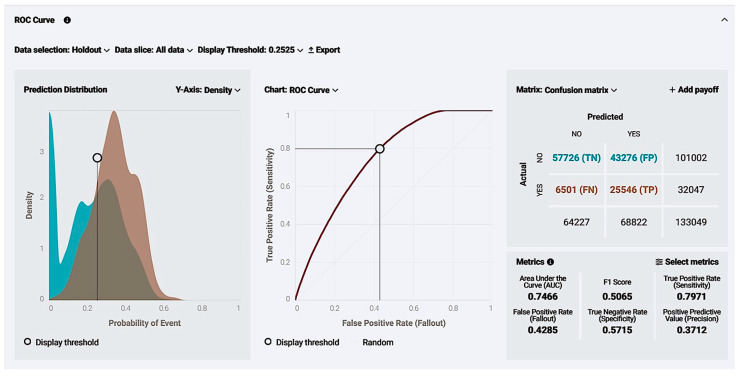
ROC curve insights for the model extracted from the “ar-2010-2018_complete features” dataset. Insights contain the prediction, distribution, which reveals that the model performance was effective as an illustration of the actual values distributed using as classification a threshold value. Additionally, metrics for the AUC score, sensitivity, specificity, etc., are displayed.

The model was further explained and evaluated by ROC curve insights. We managed to deploy a model with sufficient metrics such as the AUC score, sensitivity, and specificity, and a great quality model as the curve was growing exponentially for lower x-axis values and was distant from the random model diagonal. Additionally, the prediction distribution graph revealed that the model performance was effective as an illustration of the actual values distributed using as classification a threshold value (Figure 8). To the left of the threshold line, false predictions appear, and the right graph appears as true. The blue graph on the left correlates to the true-negative (TN) predictions, and the brown graph on the left to false negatives (FN), meaning a case with no live birth correctly identified as a “NO” in the live birth occurrence and a case with a live birth incorrectly identified as a “NO” in the live birth occurrence, respectively, while the orange graph on the right is equivalent to true positive (TP), revealing the density of cases with a live birth successfully identified as a “YES” in the live birth occurrence, noting that brown parts of the chart are mostly overlapping events. The model seems reliable with TP events, with an adequate amount in contrast with the false ones.

**Figure 9:**
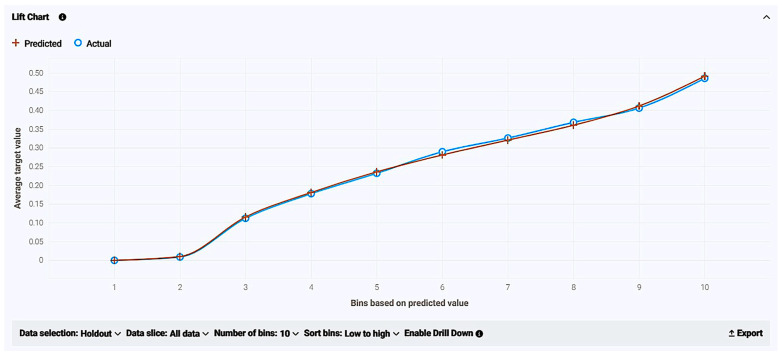
Lift chart for the model extracted from the “ar-2010-2018_complete features” dataset. The lift chart reveals reliability by presenting the average predicted probability of live birth (orange chart) along with the average proportion of live births (blue graph) for the rows from the holdout data bin.

As for model accuracy, the lift chart shown below reveals the reliability by presenting the average predicted probability of live birth (orange cross chart) along with the average proportion of live births (blue circle graph) for the rows from the holdout data bin. Both lines are extremely close to each other and crossing one another quite frequently, meaning legitimate accuracy and efficiency and no over- and/or underestimation (Figure 9).

**Figure 10:**
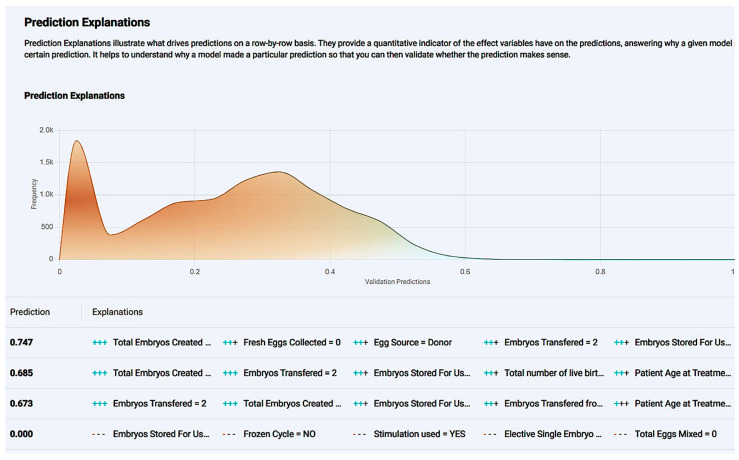
The prediction explanation offers a comprehensive description of a case’s positive prediction score and the amount of positive or negative impact each value offered in contrast with the frequency of the result.

*Prognostic Application:* The application environment offers a review of model insights explained at model registration as well as prediction options like individual predictions’ explanations with a comprehensive description of a case’s positive prediction score and the amount of positive or negative impact each value offered in contrast with the frequency of the result (Figure 10). So, one could easily understand how the model evaluates cases and validate whether a prediction is senseful. In addition, it is possible to make new predictions by adding one’s own values on impactful and important features to test and work the prediction model.

**Figure 11:**
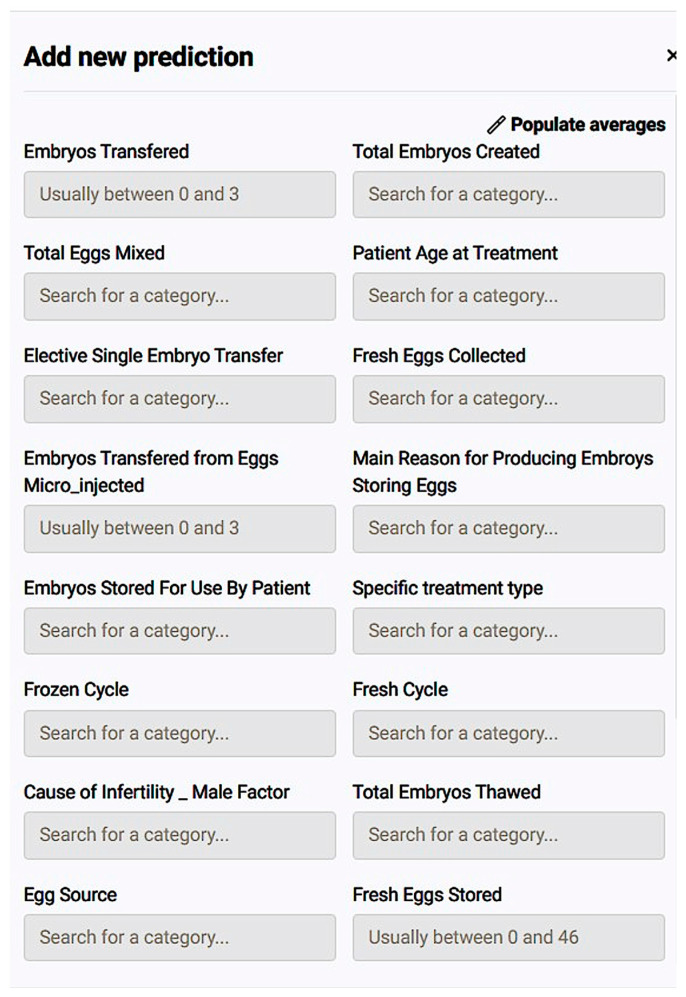
Single-case new prediction environment derived from the application development of the “ar-2020-2018_complete feature” dataset model. User interaction table containing every impactful feature with different values present in the dataset were available as options.

**Figure 12:**
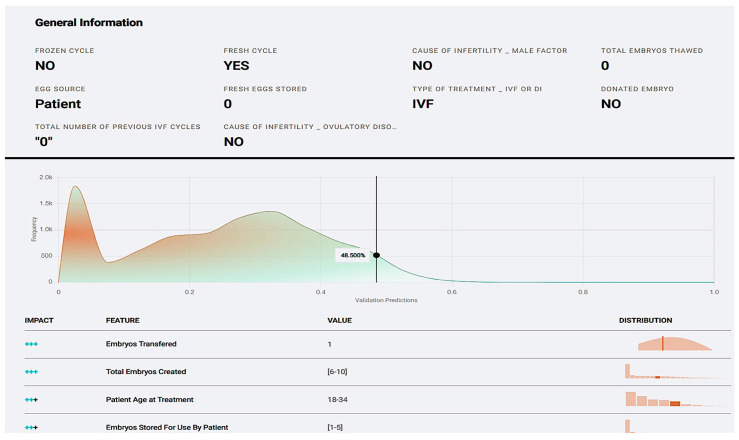
User-submitted case prediction explanation based on features from the “ar-2010-2018_complete feature” model.

As a matter of fact, we managed to submit a single case for prediction using populate averages in the questionnaire that appeared (Figure 11). The result was a prediction of the live birth occurrence at 48.5% and a preview of how each registered value affected the potential live birth (Figure 12).

**Figure 13:**
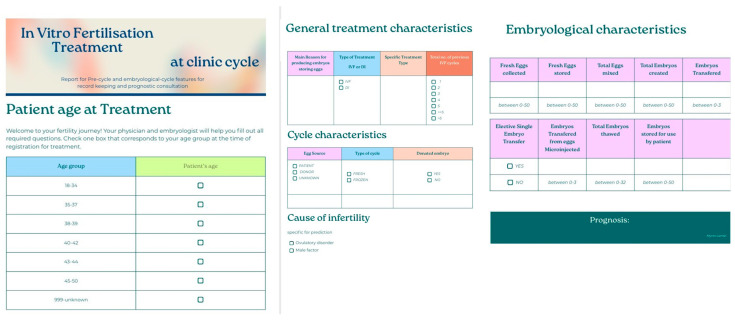
Patient-friendly questionnaire created for potential incorporation in the data registration procedure for treatments at the clinic. The questionnaire was based on the features that appeared impactful for the model developed from the “ar-2010-2018_complete features” dataset.

*Questionnaire:* To further study how such a prognostic model could be incorporated into medical everyday practice, a simple, user-friendly questionnaire, based on the important features the model requires to make a prediction, was created as a test on how a clinic could present the prognosis and treatment consultation into the treatment protocol (Figure 13).

#### 3.3.2. Modeling for “ar-2010-2018_Pre-Cycle Features”, Predictive Model for Live Birth Prognosis Based on Pre-Cycle Features That Solely Refer to Patients’ Informative Features

*Modeling:* 59 possible models were generated for the live birth occurrence target, with comprehensive autopilot and an AUC optimization metric based on informative features with stratified sampling and set for cross-validation and 20% holdout data. The highly validated and 100% sample size model was used for application development. The main model was compared to a sufficiently validated one but based on a completely different algorithm type and appeared with 64% sample size (425,756 row usage) (Table 10).

**Figure 14:**
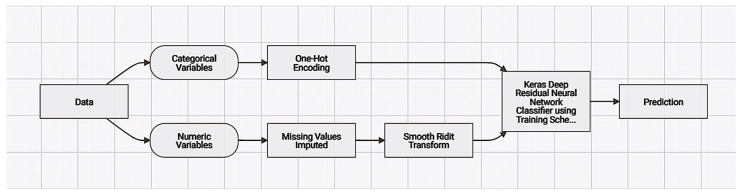
Blueprint extracted from chosen algorithm of the “ar-2010-2018_pre-cycle features” dataset modeling. Blueprint explains the dataset’s pre-processing, post-processing, and algorithm deployment steps for model development.

Model for deployment:

The initial dataset’s pre-processing, post-processing, and algorithm deployment steps are all included in the blueprint, which explains the modeling process (Figure 14). Further data processing was therefore essential for the development of the model, and the procedures are digitalized below.

**Figure 15:**
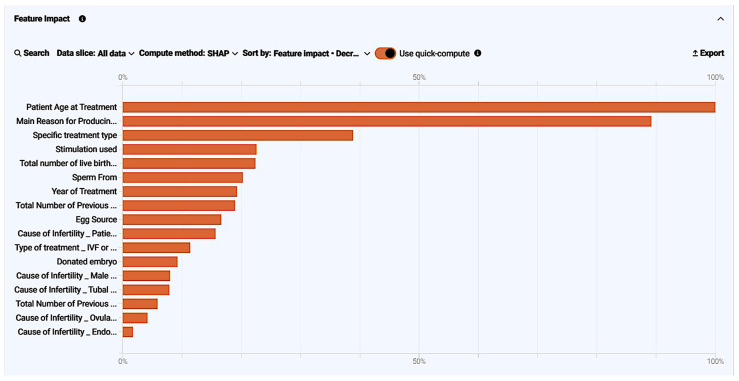
Feature impact of every feature present in the “ar-2010-2018_pre-cycle features” dataset visualized. Feature impact ranks all features based on their influence in the live birth occurrence.

The model explains which of the impactful features had a more key role in decision-making. These impacts were measured using training data. Feature impact was calculated and normalized to a 0–100 scale by the SHAP method. The findings suggest that the pre-cycle and patient-driven features with a high impact in determining live birth occurrence were: 1. patient age at treatment (normalized score = 100%); 2. the main reason for producing embryos, storing eggs (normalized score = 89.2%); 3. specific treatment type (normalized score = 38.88%); 4. stimulation used (normalized score = 22.57%); 5. total number of live births—conceived through IVF or DI (normalized score = 22.39%); 6. sperm source (normalized score = 20.25%). More specifically, the relative feature impact was demonstrated both as a chart (Figure 15) and table (Table 11) for easier analysis.

**Figure 16:**
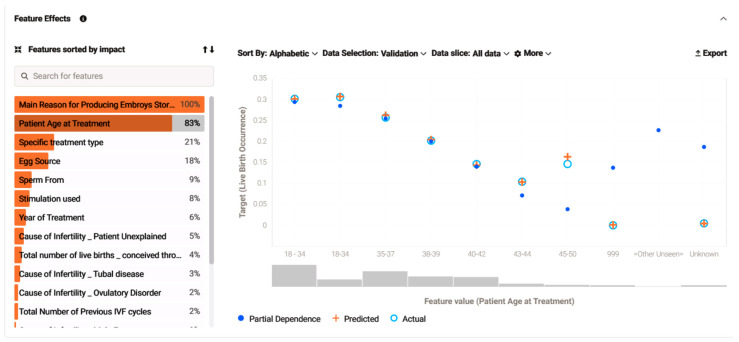
Feature effect visualized by displaying the most common values with the target resulting in a partial dependence graph line (blue dots), the average prediction for an individual feature value (orange crosses), and the actual target outcome for the individual feature values (light blue circles). In this figure, “Patient Age at treatment” feature effect is displayed. All feature effects refer to “ar-2010-2018_pre-cycle features” dataset modeling.

Addressing feature effect, the model managed to perform a calculation of how the prediction outcome can be affected by changes in each feature based on their impact. It determined the relationship between each feature individually and the target, without altering other feature values. Regarding the five features with the most significant impact, it shows that, with a decreasing effect percentage, the variables were 1. the main reason for producing embryos, storing eggs; 2. patient age at treatment; 3. specific treatment type; 4. egg source; 5. sperm from. The chart below interrelates and displays one of the most effective values (patient age at treatment) in predicting the target, resulting in a partial dependence graph line (blue dot points) explaining how changes in each unique feature regulate the result, unveiling their relationship. Simultaneously, the orange crosses calculate an average prediction for an individual feature value while still focusing on the feature selected while leaving all other features stable. The light blue circles calculate the actual target outcome for the individual feature values and compare the actual and predicted target values, helping evaluate model accuracy (Figure 16).

**Figure 17:**
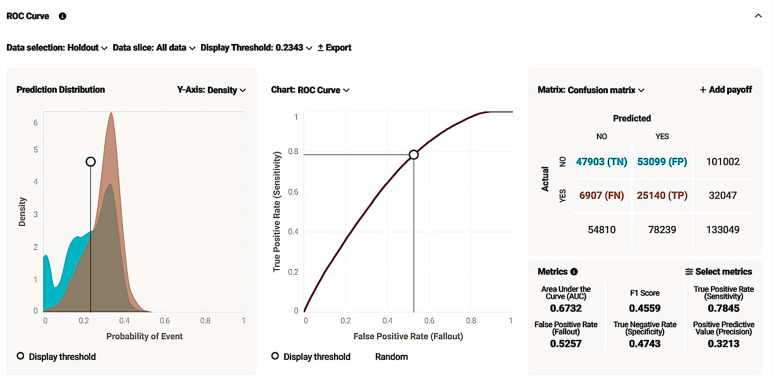
ROC curve insights for model extracted from the “ar-2010-2018_pre-cycle features” dataset. Insights contain prediction distributions, revealing that the model performance was effective as an illustration of the actual values distributed using as a classification of the threshold value. Additionally, metrics for AUC score, sensitivity, specificity, etc., are displayed.

The model is further explained and evaluated by ROC curve insights. We managed to deploy a model with sufficient metrics such as the AUC score (value over 0.5), sensitivity, and specificity, and the model was of great quality, as the curve grew exponentially for lower x-axis values and was distant from the random model diagonal. Additionally, the prediction distribution graph revealed that the model performance was effective as an illustration of the actual values distributed using as a classification of the threshold value (Figure 17). To the left of the threshold line, false predictions and the right graph appear as true. The blue graph on the left correlates to true-negative (TN) predictions, and the brown graph on the left to false negative (FN), meaning a case with no live birth correctly identified as a “NO” in live birth occurrence and a case with a live birth incorrectly identified as a “NO” in live birth occurrence, while the orange graph on the right is equivalent to true positive (TP), revealing the density of cases with a live birth successfully identified as a “YES” in live birth occurrence, noting that the brown parts of the chart are mostly overlapping events. The model seems of great reliability with TP events with an adequate amount in contrast with the false ones.

**Figure 18:**
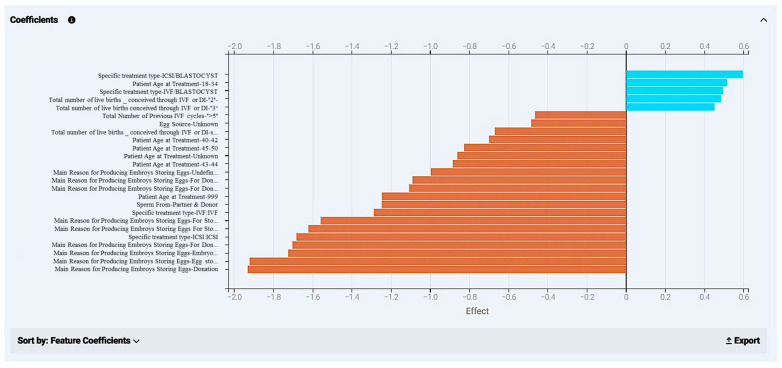
Coefficients calculated from the model development of the “ar-2020-2018_pre-cycle features” dataset. The chart displays different kinds of features with the highest impact on the final prediction. Values that appear in blue have a positive effect in the outcome and display how much they navigate a case to a positive effect (“YES” in live birth occurrence); orange bars refer to values that drive a case towards a negative outcome (“NO” in live birth occurrence).

For this model, we were able to retrieve a coefficients chart to further analyze the relative effect of distinct values of different features. Here, 25 values appear from different kinds of features with the highest impact on the final prediction (Figure 18). In blue, values appear that have a positive effect in the outcome and display how much they navigate a case to a positive effect (“YES” in live birth occurrence), while orange bars refer to values that drive a case towards a negative outcome (“NO” in live birth occurrence), respectively. With that in mind, it is easy to target case circumstances beneficial for the outcome in the future. What is important is that the positive or negative effect of values given by the model are biologically validated. Patients in the 18–34 age group, with two or three previous live births, and an ICSI treatment in the blastocyst stage are factors to search for in a case for their possible positive impact in live birth occurrence.

**Figure 19:**
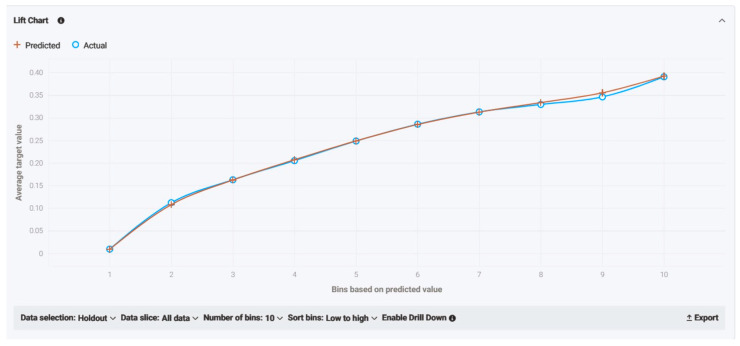
Lift chart for the model extracted from the “ar-2010-2018_pre-cycle features” dataset. The lift chart reveals reliability by presenting the average predicted probability of live birth (orange chart) along with the average proportion of live births (blue graph) for rows from the holdout data bin.

As for model accuracy, the lift chart shown below reveals the reliability by presenting the average predicted probability of live birth (orange cross chart) along with the average proportion of live births (blue circle graph) for the rows from the holdout data bin. Both lines are extremely close to each other and crossing one another quite frequently, meaning legitimate accuracy and efficiency and no over- and/or underestimation (Figure 19).

**Figure 20:**
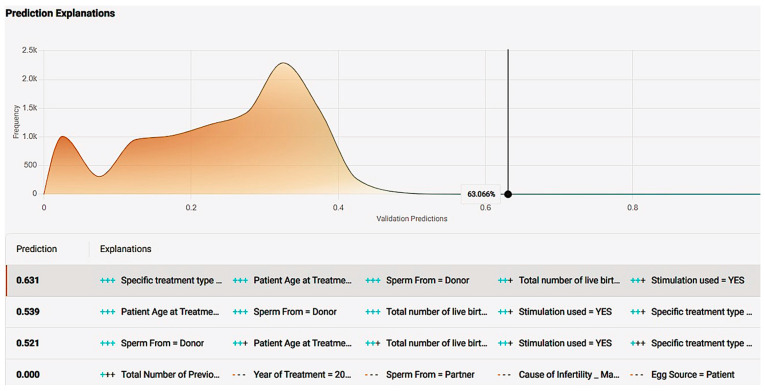
Prediction explanation offering a comprehensive description of a case’s positive prediction score and the amount of positive or negative impact each value offered in contrast with the frequency of the result.

**Figure 21:**
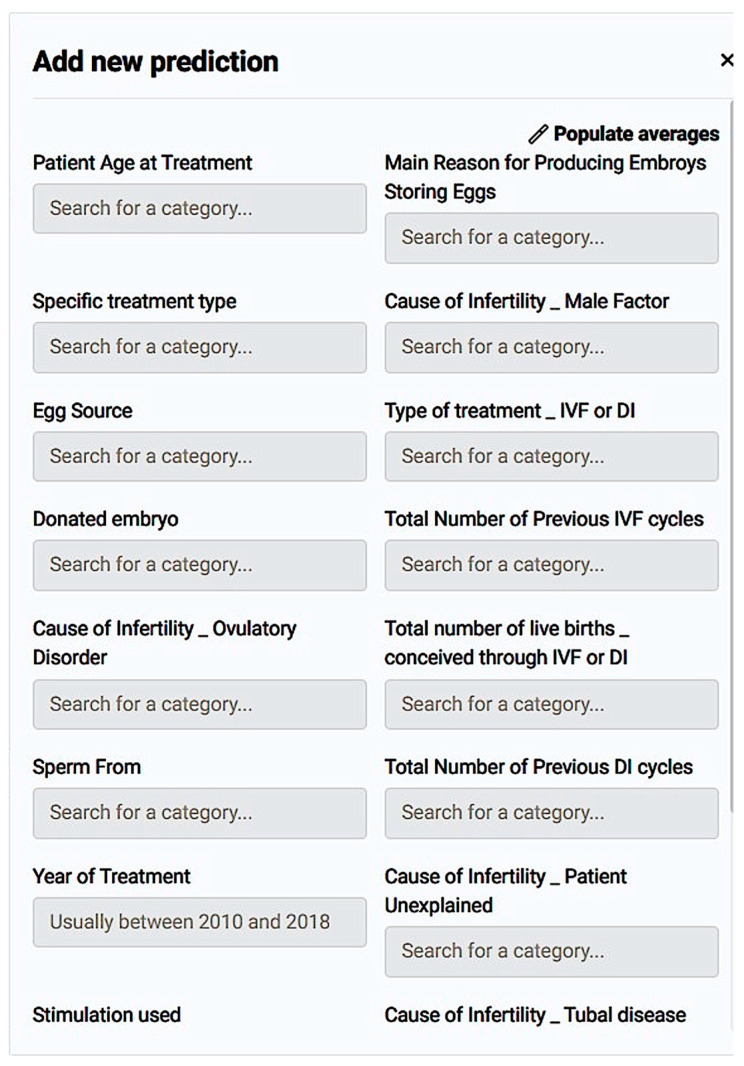
Single-case new prediction environment derived from the application development of the “ar-2020-2018_pre-cycle features” dataset model. User interaction table containing every impactful feature with different values present in the dataset were available as options.

*Prognostic Application:* The application environment offers a review of model insights explained at model registration as well as prediction options like individual predictions’ explanations with a comprehensive description of a case’s positive prediction score and the amount of positive or negative impact each value offered in contrast with the frequency of the result (Figure 20). So, one could easily understand how the model evaluates cases and validate whether a prediction is senseful. In addition, it is possible to make new predictions by adding one’s own values on impactful and important features to test and work the prediction model (Figure 21).

**Figure 22:**
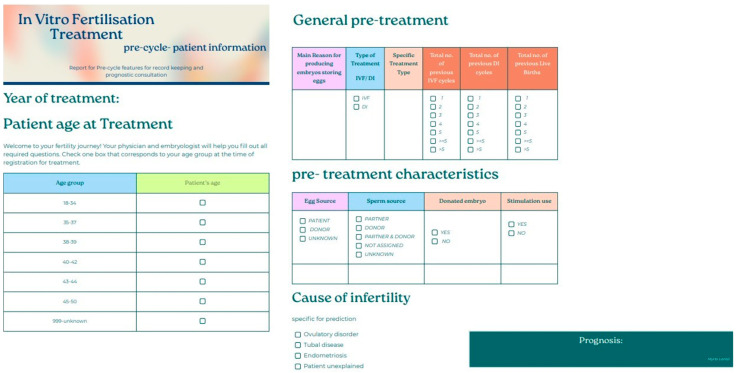
Patient-friendly questionnaire created for potential incorporation in the data registration procedure for treatments at the clinic. The questionnaire was based on features that appeared impactful for the model developed from the “ar-2010-2018_pre-cycle features” dataset.

*Questionnaire:* To further study how such a prognostic model could be incorporated into medical everyday practice, a simple, user-friendly questionnaire, based on the important features the model requires to make a prediction, was created as a test on how a clinic could present the prognosis and treatment consultation into the treatment protocol (Figure 22).

## 4. Discussion

### 4.1. Thoughts on Complete Fertility Data Study Analysis

The guidelines extrapolated from this data, along with the predictive model questionnaires serve as foundational framework, albeit they overlook the interplay and significance of patient-oriented factors. A myriad of potential barriers to successful child-bearing warrant consideration and create a gap in the documentation of case-characteristics. Specifically, pre-cycle and pre-treatment features such as the gynecological history of the woman undergoing treatment should be meticulously recorded, encompassing parameters such as cycle regularity, length, and menstrual flow, as those could offer insights and be beneficial for improving and personalizing implantation protocols. Furthermore, additional characteristics could be used as biomarkers and prognostic indicators such as hormonal profiles, as they can exert an impact on stimulation induction and adjunctive in IVF therapy. Similarly, reaction to stress, anxiety levels, and potential chronic inflammation could alter the gestational environment and pregnancy receptiveness [49,50]. Moreover, the overlook of dietary and lifestyle habits as well as body mass index (BMI) could enhance comprehensiveness of each case as all factors have been associated with variations in reproductive outcomes [51,52].

From a bioethical perspective, it is imperative to acknowledge the implications of establishing a standardized successful fertility model based on categorized patient characteristics. This approach would discourage, discriminate, and limit women from pursuing fertility, particularly when the circumstances do not conform with those idealized. Consequently, such a perspective could perpetuate inequality in reproductive health care. Additionally, this fails to recognize how fertility and gestation are multifaceted and often aleatory incidents. Indeed, a successful outcome has been observed in diverse factor combinations, underscoring the uniqueness and complexity of childbirth. Therefore, the principle upheld should be that every individual seeking fertility treatment has the right to receive assurance on pursuing childbearing and make informed choices, irrespective of their background or profile, as there should not be boundaries to parenthood.

### 4.2. Future Research—Questionable Results

Further investigation prompts inquiries into the factors contributing to better live birth prognosis in the 45–50-year age bracket in comparison to the 40–44-year bracket. Moreover, understanding the gynecological state of a 43–44-year-old patient is crucial for elucidating the potential hindrance of a successful gestation. It could easily be hypothesized that the majority of 45–50-year-old patients may have opted for egg donation, given the maternal-age-related diminish in success rates. However, the use of donor oocytes was comparatively analyzed in the 43–44 and 45–50 age groups. Both cohorts exhibited a higher prevalence of cases conducted with the utilization of donors’ oocytes, indicating that the lower percentage of successful gestations in the 43–44 age bracket cases may have been attributable to the inherent gynecological factors of the carrier. Thus, an essential inquiry arises regarding the disparities between women in their early 40s and women in their late 40s and early 50s.

To delineate the disparities between those age groups, attention should be delved into the physiological events occurring in a woman’s body across different times of their lives. Fertility, being intrinsically linked to age, is extensively elucidated and the progressive changes meticulously clarified [53]. The zenith of reproductive capacity occurs during the third decade of a woman’s life, subsequent years are followed by gradual fertility decline, eventually preceding to menopause [53]. Menopause is defined as the absence of menstruation and inability to achieve pregnancy because of the cessation of ovarian follicular activity and is diagnosed by a 12-month amenorrhea from natural causes [54,55]. This biological milestone occurs in a woman’s early 50s with a mean age of 51, while the years around this incidence are referred to as perimenopause [56]. Perimenopause or menopausal transition begins with the first onset of menstrual irregularity and is marked by 1 year of amenorrhea, thereby defining the final menstrual period (FMP). It usually spans five years—although it can vary from two to eight years—predominantly aligning with the 45–50 age bracket [54,56]. To classify women’s menstrual state, the Stages of Reproductive Aging (STRAW) criteria were developed, providing guidelines clustering women into stages based on age and menstrual status—with menopause denoted as stage 0 [57].

The distinction between women aged 40–44 and 45–50 years lies in their respective stages of reproductive aging. Women undergoing the treatment aged 45–50 are more likely to be classified under STRAW stage (−1), referred to as early and late perimenopause, while those aged 40–44 are experiencing their late reproductive age, designated as stage (−3) [58]. In exploring factors affecting treatment outcome in 43–44-year-old patients, it is crucial to recognize characteristics associated with late reproductive age stage. Menstrual cycles generally remain normal, albeit hormonal markers such as FSH levels exhibit irregular fluctuations throughout the 28-day biological calendar; AMH, AFC, and inhibin B levels begin to decline variably in cycle stages; estradiol levels mainly remain stable; and no significant somatic symptoms are visible [59]. Conversely, perimenopausal women often experience missed menstrual cycles for a month or more, along with variations in cycle length [57,60]. FSH and LH levels steadily elevate, AMH decreases significantly, and progesterone and E2 levels decline during the transition period [57].

Given the widespread utilization of hormonal medication in IVF protocols, it is conceivable that the inconsistent endocrine profile of women in late reproduction age may exhibit unexpected challenges compared to the more predictable hormonal profiles of perimenopausal and menopausal women. The unpredictable responses to medication and hormonal induction ART protocols in late-reproductive-aged women underscore the need for personalized and tailored hormone therapies. In contrast, perimenopausal and menopausal women have a more predictable and defined hormonal profile and could benefit from a standardized hormonal scheme specific to IVF treatment.

This divergence in the—mainly asymptomatic—reproductive status of 40–44-year-old women, coupled with stimulation induction, could hypothetically create a less receptive environment for successful embryo implantation. Therefore, the clinic’s data findings underscore the importance of further research into the menstrual characteristics of the ART patients, particularly those beyond the peak fertility stage, with the aim of creating a promising IVF treatment personalized under diverse circumstances. Nevertheless, while data analysis confirms correlations between case factors and treatment outcomes, it is imperative to acknowledge the haphazardness of childbirth and the enigmatic nature behind reproduction.

However, revolutionary knowledge has emerged indicating that the process of aging is not a gradual decline but rather occurs in spurts along two distinct timelines. One appears at the age of 44, and one at 60 years old. Notably, the rate of aging between the two main bursting sites is slower. Aging encompasses all biological systems in the body, including the reproductive system, which is undoubtedly affected. Further investigation is required to ascertain whether the aging process that commences at 44 has a greater impact on fertility in women, compared to those who reach the subsequent aging stage of 60 [61].

### 4.3. Success Rates and Epigenetic Impact

Being cognizant of the extensive improvement of the IVF procedure, relatively to its recent establishment—in terms of medical practice—and various clinical trials being run at the same time IVF is performed, we were driven to calculate the growth of the success rate during the past decades. The fortunate outcome is characterized by a live birth and the occurrence refers to the years 1991 through 2018.

While a significant rise in successful treatment—of the order of a 15% increase—in the past 30 years ascertains the continuous progress of the IVF method, the unceasing rise seems to have a decrease in its rate when, in fact, a stabilization in recent years is noted (Table 12). Additionally, we could suggest that there have been no major changes in the last 10 years since the Human Fertilization amd Embryology Authority (HFEA) supports that a rate of 24% is also calculated for the year 2019. These findings are particularly intriguing further research of a potential stagnation in the effectiveness of the procedure. Correspondingly, according to HFEA, pregnancy rates follow related fluctuations.

The pursuit of serving one-sixth of the global population suffering from subfertility involves ART’s endeavor to serve them. Amid the fertility issues, considerable improvements have been made in the IVF protocol. Hormonal medications have become safer and gentle, practices involving cryopreservation are established, and surrogates’ and donors’ inclusion is practiced. The pertinent question persists: What further refinements could be made?

Specifically, the query revolves on whether there is still room for innovation in the laboratory procedures of in vitro fertilization (IVF), or if the dish handling is already at an optimal level. Should the reset button, in further increasing live births, be searched in other aspects of the treatment? Since the main stabilization in rates appears to be seen in the 2010s, which coincides with novel hormonal medications being used and laboratory procedure being refined, the pace of everyday life might be epigenetically linked to the phenomenon. In this context, the environment, stressful lifestyle, workload, and modern-life shifts in diet along with the emotional and psychological impact of the treatment could interfere with the mother’s hormonal and biochemical state. In turn, these states are crucial for the preparation for egg retrieval, embryo transfer, and gestation.

Furthermore, oocyte and embryo development are affected by such epigenetic changes. Starting in gametogenesis, all epigenetic marks of a differentiated cell are deleted in the primordial germ cell stage to prepare for reprogramming. Theoretically, in early embryogenesis and during fertilization, parental DNA undergoes rapid demethylation in a process called extensive nuclear reprogramming, meaning the epigenetic state of the gametes is reset before implantation where epigenetic changes happen anew to create totipotent cells. However, in a more in-depth look, methylation is removed from the paternal DNA after fertilization, while maternal DNA gradually decreases its methylation marks during replication. These epigenetic changes are regulate gene expression through imprinting in embryo development since they are preserved on the maternal gamete and inherited to the offspring. It is important to mention that mtDNA, mitochondria, and miRNAs are maternally inherited, amplifying the significance of the mother’s epigenome and genome state [62,63].

Respectively, the embryonic genome is highly demethylated in the early blastocyst age. Subsequently, methylation patterns occur anew, and epigenetic mechanisms are reestablished during further fetal development. Many of these epigenetic profiles remain imprinted after birth and during the lifetime of the offspring, pointing to the significance of the environment that encloses the fetus in regular development. It is possible to hypothesize that the outcome of the IVF treatment—apart from a safe and successful lab operation—is highly dependent on the mother and/or carrier. It is apparent that the idealization of the direct environment of oocytes and embryos should be carefully studied and interrelated with the ART treatment [63].

### 4.4. Evaluation of Predictive Machine Learning Models

The outcomes of our predictive model attempt seemed promising and enhanced the idea of AI integration in the clinical workflow, helping to refine fertility treatment strategies and aid in decision-making, thus raising further questions about the incorporation of a machine learning driven predictive model. As a successful subfertility treatment, the overall modeling and application design demonstrated coherence, accuracy, and functionality in predicting the occurrence of a live birth. However, putting the model into practice calls for ongoing algorithm testing, validation, retraining, and additional data processing to prevent bias in the training set. For a prognostic tool of this kind to be smoothly integrated into the treatment process, it must be improved and tested for additional, more thorough registered data. Furthermore, the various prognostic model designs draw attention to the necessity of individualized, woman-patient-driven care. Many predictive tools concern embryological and cycle features, while the additional data processing for including solely pre-cycle features showed positive results and feasible design.

Interestingly, the predictive models showcased the cause of infertility as an important factor, present in both feature registration questionnaires for prediction. Comparing it to the bioinformatic analysis, causes like ovulatory disorder, male factor, tubal disease, or unexplained reasons were among the features that seemed impactful in both our statistical analysis and AI data exploration. Subsequently, when studying the coefficients in the pre-cycle analysis, we were able to notice that the patients’ age groups had different negative impacts on the outcome. More specifically, the age bracket of 43–44-year-old patients presented the greater negative impact in the positive live birth outcome, then came 45–50 age group, and the least negative impact was showcased by the 40–42 age group. Our log-minus-log plot suggested that the age group of 43–44 years presented the biggest reduction in probability of live birth, while the 45–50 age group had a better prognosis. Subsequently, these findings lead to validation of the effectiveness and accuracy of the predictive models, triggering the interest in the establishment of such novelties.

Our analysis demonstrates that, with a high degree of accuracy, it is possible to construct a robust and clinically applicable model exclusively on pre-cycle features of interest. The utilization of data available prior to initiating a new IVF cycle, encompassing cardiovascular history, age, endocrine markers, and previous clinic outcomes, enabled our pre-cycle model to demonstrate high levels of competitive prediction performance. This has clinical importance, since it facilitates outcome prediction and patient counseling without initiating a further treatment cycle, thereby saving time and emotional and financial cost as well as saving costs. Concurrently, the comprehensive model encompassing both embryological and gynecological characteristics continues to demonstrate enhanced performance and improved predictive capability. However, the predictive capacity of the pre-cycle-only model underscores the potential for acquiring valuable information well in advance of the initiation of clinical interventions and presents a very applicable questionnaire in everyday practice, in contrast with more complex existing models.

A nonlinear age pattern that defies standard assumptions was also revealed by the data and shifts the balance in the predicting pattern of our model. Specifically, female patients aged 43–44 had poorer predictive results than those aged 45–50. This finding suggests the potential for this age group to constitute an exceptional clinical subgroup, influenced by physiological, behavioral, or treatment-related conditions that disrupt the typical decline curve. The identification and inquiry into this irregularity may result in the development of more age-specific and personalized predictive strategies that have not yet been presented.

## 5. Conclusions

Predictive models are becoming both more prevalent and more ubiquitous in assisted reproduction, providing clinicians with useful tools for predicting the likelihood of treatment success and improving patient counselling and clinical decision-making. However, the intrinsic complexity of in vitro fertilization (IVF) protocols, including biological diversity, underlying pathologies, and patient-specific conditions, decreases the predictability and generalizability of these models. As shown in this work, although when trying to find the most statistically significant predictor of IVF outcomes, age is always in the first place, but its explanatory capacity is often overstated. This so-called “age paradox” exposes the gulf between population-level predictions based on age and individual-level variability, highlighting the importance of further understanding the physiology and complexity of childbearing individuals.

Such findings highlight the necessity of creating greater comprehensive and integrative predictive models outside of the traditional demographic and clinical characteristics. Future directions should include the addition of emerging biomarkers, genetic and epigenetic information, immunologic profiles, and psychosocial variables for a more robust representation of the multifactorial dimensions of fertility. Yet at the same time, the intensely personal and emotionally fraught process of trying to conceive ought not to be distilled into statistical probabilities. Childbearing is a biological process, but simultaneously, a deeply personal and emotionally charged journey that should not be reduced to statistical probabilities. As such, predictive models must serve not only scientific precision but also consultation and compassionate care, recognizing the unique complexity and significance of the path to parenthood.

## Figures and Tables

**Table 1:** Dataset insights, number of features included, and number of final cases used. Both datasets include all cases between 2010 and 2018 after wrangling. Dataset named “ar-2010-2018_complete features” refers to pre-cycle and embryological, in-cycle characteristics; dataset “ar-2010-2018_pre-cycle features” contains features relevant to pre-treatment.

Datasets	Features	Rows
IVF PREDICTION MODEL 2010–2018 complete features	30	665.244
IVF PREDICTION MODEL 2010–2018 pre-cycle features	18	665.244

**Table 2:** Descriptive statistics of various characteristics in fertilization procedure regarding the embryological parameters. The mean value, standard deviation, and minimum and maximum values displayed.

	Mean	SD	Mdn	Min–Max
Fresh Eggs Collected	7.3000	7.1000	6	0–50
Fresh Eggs Stored	0.0000	0.7000	0	0–46
Total Eggs Mixed	6.5000	6.4000	6	0–50
Eggs Mixed With Partner Sperm	6.3000	6.3000	5	0–50
Eggs Mixed With Donor sperm	0.4000	2.1000	0	0–50
Total Embryos Created	4.3000	4.6000	3	0–49
Eggs Micro-injected	2.8000	4.9000	0	0–50
Embryos from Eggs Micro-injected	1.9000	3.5000	0	0–43
Total Embryos Thawed	0.4000	1.3000	0	0–32
Embryos Transfered	1.5000	0.9000	2	0–4
Embryos Transfered from Eggs Micro-injected	0.6000	0.9000	0	0–4
Embryos Stored For Use By Patient	0.9000	2.5000	0	0–48
Embryos (from Eggs Micro-injected) Stored For Use By Patient	0.4000	1.5000	0	0–43

**Table 3:** Differences in age (patient, egg, sperm donor) vs. live birth occurrence.

		Non-Live Birth	Live Birth	*p*-Value
Patient Age at Treatment	18–34	0.4540	55.30%	<0.001
	35–37	0.2230	23.90%	
	38–39	0.1380	11.20%	
	40–42	0.1280	6.90%	
	43–44	0.0370	1.40%	
Egg Donor Age at Registration	45–50	0.0190	1.30%	<0.001
	<=20	0.0210	1.90%	
	21–25	0.1380	15.30%	
	26–30	0.32.50	34.70%	
	31–35	0.5010	46.70%	
	36–40	0.0160	1.50%	
Sperm Donor Age at Registration	<=20	0.0950	7.70%	<0.001
	21–25	0.3140	28.00%	
	26–30	0.2270	23.00%	
	31–35	0.1650	17.60%	
	36–40	0.1290	15.00%	
	41–45	0.0690	8.50%	
	>45	0.0010	0.20%	

**Table 4:** Differences in history and procedure vs. live birth occurrence.

		Non-Live Birth	Live Birth	*p*-Value
Total Number of Previous cycles (Both IVF & DI)	0–1	60.20%	66.20%	<0.001
	2+	39.80%	33.80%	
Total number of previous pregnancies (Both IVF & DI)	0	84.40%	80.90%	<0.001
	1+	15.60%	19.10%	
Type of treatment	IVF	81.50%	92.30%	<0.001
	DI	18.50%	7.7%	
Elective Single Embryo Transfer	No	87.50%	76.70%	<0.001
	Yes	12.50%	23.30%	
Egg Source	Patient	95.50%	94.10%	<0.001
	Donor	4.50%	5.90%	
Sperm From	Partner	77.10%	86.20%	<0.001
	Donor	22.90%	13.80%	

**Table 5:** Treatment cycle, stimulation used vs. live birth occurrence.

		Non-Live Birth	Live Birth	*p*-Value
Cycle	Fresh	0.7890	79.30%	<0.001
	Frozen	0.2110	20.70%	
Stimulation used	No	0.3120	27.10%	<0.001
	Yes	0.6880	72.90%	

**Table 6:** Differences in various characteristics in fertilization procedure vs. live birth occurrence.

	Non-Live Birth	Live Birth	*p*-Value
Total Eggs Mixed	6.08 ± 6.33	7.98 ± 6.25	<0.001
Eggs Mixed With Partner Sperm	5.83 ± 6.26	7.81 ± 6.28	<0.001
Eggs Mixed With Donor sperm	0.32 ± 1.97	0.48 ± 2.42	<0.001
Total Embryos Created	3.87 ± 4.48	5.56 ± 4.62	<0.001
Embryos Transfered	1.45 ± 0.97	1.80 ± 0.62	<0.001

**Table 7:** Cause of infertility vs. live birth occurrence.

		Non-Live Birth	Live Birth	*p*-Value
Tubal disease	No	83.2%	83.8%	<0.001
	Yes	16.8%	16.2%	
Ovulatory Disorder	No	89.6%	87.1%	<0.001
	Yes	10.4%	12.9%	
Male Factor	No	50.3%	52.7%	<0.001
	Yes	49.7%	47.3%	
Patient Unexplained	No	75.7%	72.8%	<0.001
	Yes	24.3%	27.2%	
Endometriosis	No	94.6%	94.0%	<0.001
	Yes	5.4%	6.0%	
Partner Sperm Concentration	No	85.9%	92.5%	<0.001
	Yes	14.1%	7.5%	

**Table 8:** Model algorithm information for the “ar-2010-2018_complete features” dataset.

	Model for Deployment:		
eXtreme Gradient Boosted Trees Classifier with Early Stopping (Learning Rate = 0.02)			
Training Scores: AUC		Training Settings	
Validation	0.7474	Training feature list	Informative features- Leakage removed
Cross-validation	0.747	Training sample size	100% (655,244 rows)
Holdout	0.7466		

**Table 9:** Feature impact of every feature present in the “ar-2010-2018_compete features” dataset. Normalized score: relative importance. Feature impact ranks all features based on their influence in the live birth occurrence.

Feature Name	Relative Importance
Embryos Transfered	1
Patient Age at Treatment	0.248421339
Total Embryos Created	0.173121607
Embryos Stored For Use By Patient	0.160096191
Main Reason for Producing Embryos–Storing Eggs	0.097558912
Fresh Eggs collected	0.089620152
Year of treatment	0.086912603
Elective Single Embryo Transfer	0.078173214
Total number of live births _ conceived through IVF or DI	0.071971854
Total Number of Previous IVF cycles	0.071772142
Egg Source	0.047395769
Specific treatment type	0.046299934
Total Eggs Mixed	0.042548409
Embryos Transfered from Eggs Micro injected	0.03432578
Stimulation used	0.031850656
Total Embryos Thawed	0.024764674
Frozen Cycle	0.023717849
Cause of Infertility_ Tubal disease	0.019816558
Fresh Cycle	0.017667274
Cause of Infertility _ Male Factor	0.017281755
Sperm From	0.01650989S
Type of treatment_ IVF or DI	0.012494923
Cause of Infertility _ Ovulatory Disorder	0.010995262
Fresh Eggs Stored	0.008373111
Cause of Infertility _ Patient Unexplained	0.005891879
Total Number of Previous DI cycles	0.005842854
Cause of Infertility _ Endometriosis	0.003256256
Donated embryo	0.000734093

**Table 10:** Model algorithm information for the “ar-2010-2018_pre-cycle features” dataset.

Keras Deep Residual Neural Network Classifier Using Training Schedule (3 Layers: 512, 64, 64 Units)			
Training Scores: AUC		Training Settings	
Validation	0.6725	Training feature list	Informative features
Cross-Validation	0.672	Training sample size	100% (655,244 rows)
Holdout	0.6732		

**Table 11:** Feature impact of every feature present in the “ar-2010-2018_pre-cycle features” dataset. Normalized score: relative importance. Feature impact ranks all features based on their influence in the live birth occurrence.

Feature Name	Relative Importance
Patient Age at Treatment	1
Main Reason for Producing Embroys Storing Eggs	0.8920162731263795
Specific treatment type	0.3887550025854647
Stimulation used	0.22568717745283595
Total number of live births_ conceived through IVF or DI	0.22391198318149877
Sperm From	0.20252134584065593
Year of Treatment	0.1928651226537317
Total Number of Previous IVF cycles	0.18957181358844258
Egg Source	0.16616890864503883
Cause of Infertility Patient Unexplained	0.1564999572046616
Type of treatment_ IVF or DI	0.11384410152058551
Donated embryo	0.092351681
Cause of Infertility_Male Factor	0.07976977
Cause of Infertility_Tubal disease	0.078500847
Total Number of Previous DI cycles	0.058750517288136066
Cause of Infertility_Ovulatory Disorder	0.041770416
Cause of Infertility_Endometriosis	0.017128636722770634

**Table 12:** Success rates of IVF treatments at the clinic throughout the years 1991–2018. Live birth occurrence is assigned as successful treatment.

Year of Treatment	Live Birth Occurrence (%)	Total Live Birth Occurrence (%)
1991–1994	9.4396	20.1467
1995–1999	15.0564	
2000–2004	18.2843	
2005–2009	22.0873	
2010–2014	24.0658	
2015–2016	24.0236	
2017–2018	24.1858	

## Data Availability

The data presented in this study are available on request from the corresponding author.

## Supplementary Materials

The following supporting information can be downloaded at: https://www.mdpi.com/article/10.3390/biology14050556/s1, Table S1: Dataset insights features included. Both datasets include all cases between 2010–2018 after wrangling. Dataset named “ar-2010-2018_complete features” lists all pre-cycle, embryological and in-cycle characteristics, Dataset “ar-2010-2018_pre-cycle features” contains features relevant to pre-treatment.

## Abbreviations

The following abbreviations are used in this manuscript:

IVF	In vitro fertilization
ART	Assisted reproductive technology
DI	Donor insemination
COS	Controlled ovarian stimulation
OHS	Ovarian hyperstimulation syndrome
ICSI	Intracytoplasmic sperm injection
IUI	Intrauterine insemination
GIFT	Gamete intrafallopian transfer
ZIFT	Zygote intrafallopian transfer
FSH	Follicle-stimulating hormone
LH	Luteinizing hormone
CC	Clomifene
hcG	Human chorionic gonadotropin
GDF9	Growth Differentiation Factor 9
BMP	Bone Morphogenetic Protein
GnRha	Gonadotropin-Releasing Hormone Agonist
rFSH	Recombinant Follicle-Stimulating Hormone
AMH	Anti-mullerian hormone
DHEA	Dehydroepiandrosterone
AFC	Antral follicle count
IVM	In vitro maturation
IVA	In vitro activation
IVG	In vitro gametogenesis
CNP	C-type Natriuretic Peptide
WHO	World Health Organization
DMEM	Dulbecco’s Modified Eagle Medium
HSA	Human serum albumin
LBR	Live birth rate
PR	Pregnancy
AAs	Amino acids
PGT	Preimplantation genetic testing
ET	Embryo transfer
PCOS	Polycystic ovary syndrome
POI	Primary ovarian insufficiency
PID	Pelvic inflammatory disease
SGA	Small for gestational age
TSH	Thyroid-stimulating hormone
FGR	Fetal growth restriction
CPR	Cumulative pregnancy rate
CLBR	Cumulative live birth rate
AI	Artificial intelligence
PM	Precision medicine
EVE	Ex vivo uterine environment
ML	Machine learning
DL	Deep learning
ANN	Artificial neural network
FDA	Food and Drug Association
AUC	Area under the curve
SHAP	SHapley Additive exPlanations
ROC	Receiver operating characteristic
BMI	Body mass index
FMP	Final menstrual period
STRAW	Stages of Reproductive Aging
E2	Estradiol
HFEA	Human Fertilization and Embryology Authority
mtDNA	Mitochondrial DNA
miRNA	MicroRNA
