# Supplementary material for: Bioinformatic Analysis of Complex In Vitro Fertilization Data and Predictive Model Design Based on Machine Learning: The Age Paradox in Reproductive Health
Source: Biology (Basel). 2025 May 16;14(5):556. doi: 10.3390/biology14050556 (PMC12108729; doi:10.3390/biology14050556)
Supplement: Supplementary file 1 [file biology-14-00556-s001.zip › biology-3599565-supplementary.pdf]

Article

Bioinformatic Analysis of Complex In Vitro Fertilization Data & Predictive Model Design Based on Machine Learning; The Age Paradox in Reproductive Health.

Myrto A. Lantzi[1,2], Eleni Papakonstantinou[1,2], Dimitrios Vlachakis[1,2,3]*

[1] Laboratory of Genetics, Department of Biotechnology, School of Applied Biology and Biotechnology, Agricultural University of Athens, 11855 Athens
[2] University Research Institute of Maternal and Child Health and Precision Medicine, School of Medicine, National and Kapodistrian University of Athens, 11527 Athens, Greece
[3] Algorithms and Bioinformatics Group, Informatics Department, Faculty of Natural, Mathematical & Engineering Sciences, Strand Campus, WC2R 2LS, King's College London.
* Correspondence: Dr Dimitrios Vlachakis, Laboratory of Genetics, Department of Biotechnology, School of Applied Biology and Biotechnology, Agricultural University of Athens, 75 Iera Odos, 11855 Athens, Greece E-mail: dimitris@aua.gr

Supplementary Material

Supplementary Table S1

IVF PREDICTION MODEL 2010-2018 complete features	IVF PREDICTION MODEL 2010-2018 pre-cycle features
Live Birth Occurrence	Live Birth Occurrence
Patient Age at Treatment	Patient Age at Treatment
Total Number of Previous IVF cycles	Total Number of Previous IVF cycles
Total Number of Previous DI cycles	Total Number of Previous DI cycles
Total number of live births - conceived through IVF or DI	Total number of live births - conceived through IVF or DI
Cause of Infertility - Tubal disease	Cause of Infertility - Tubal disease
Cause of Infertility - Ovulatory Disorder	Cause of Infertility - Ovulatory Disorder
Cause of Infertility - Male Factor	Cause of Infertility - Male Factor
Cause of Infertility - Patient Unexplained	Cause of Infertility - Patient Unexplained
Cause of Infertility - Endometriosis	Cause of Infertility - Endometriosis
Main Reason for Producing Embroys Storing Eggs	Main Reason for Producing Embroys Storing Eggs
Stimulation used	Stimulation used
Donated embryo	Donated embryo
Type of treatment - IVF or DI	Type of treatment - IVF or DI
Specific treatment type	Specific treatment type
Elective Single Embryo Transfer	Egg Source
Egg Source	Sperm From
Sperm From	Year of Treatment
Fresh Cycle	
Frozen Cycle	
Fresh Eggs Collected	
Fresh Eggs Stored	
Total Eggs Mixed	
Total Embryos Created	
Embryos Transfered	
Total Embryos Thawed	
Embryos Transfered from Eggs Micro-injected	
Embryos Stored For Use By Patient	
Year of Treatment	
Number of Live Births	

Table S1. Dataset insights features included. Both datasets include all cases between 2010-2018 after wrangling. Dataset named "ar-2010-2018_complete features" lists all pre-cycle, embryological and in-cycle characteristics, Dataset "ar-2010-2018_pre-cycle features" contains features relevant to pre-treatment.
